# Angular momentum holography via a minimalist metasurface for optical nested encryption

**DOI:** 10.1038/s41377-023-01125-2

**Published:** 2023-03-28

**Authors:** Hui Yang, Peng He, Kai Ou, Yueqiang Hu, Yuting Jiang, Xiangnian Ou, Honghui Jia, Zhenwei Xie, Xiaocong Yuan, Huigao Duan

**Affiliations:** 1 National Research Center for High-Efficiency Grinding, College of Mechanical and Vehicle Engineering, Hunan University, Changsha, 410082 China; 2 Nanophotonics Research Center, Shenzhen Key Laboratory of Micro-scale Optical Information Technology, Institute of Microscale Optoelectronics, Shenzhen University, Shenzhen, 518060, Guangdong China; 3 Institute of Precision Optical Engineering, School of Physics Science and Engineering, Tongji University, Shanghai, 200092 China; 4 Greater Bay Area Institute for Innovation, Hunan University, Guangzhou, 511300, Guangdong Province China

**Keywords:** Nanophotonics and plasmonics, Metamaterials

## Abstract

Metasurfaces can perform high-performance multi-functional integration by manipulating the abundant physical dimensions of light, demonstrating great potential in high-capacity information technologies. The orbital angular momentum (OAM) and spin angular momentum (SAM) dimensions have been respectively explored as the independent carrier for information multiplexing. However, fully managing these two intrinsic properties in information multiplexing remains elusive. Here, we propose the concept of angular momentum (AM) holography which can fully synergize these two fundamental dimensions to act as the information carrier, via a single-layer, non-interleaved metasurface. The underlying mechanism relies on independently controlling the two spin eigenstates and arbitrary overlaying them in each operation channel, thereby spatially modulating the resulting waveform at will. As a proof of concept, we demonstrate an AM meta-hologram allowing the reconstruction of two sets of holographic images, i.e., the spin-orbital locked and the spin-superimposed ones. Remarkably, leveraging the designed dual-functional AM meta-hologram, we demonstrate a novel optical nested encryption scheme, which is able to achieve parallel information transmission with ultra-high capacity and security. Our work opens a new avenue for optionally manipulating the AM, holding promising applications in the fields of optical communication, information security and quantum science.

## Introduction

Holography, invented by Gabor, provides an approach for recording and reconstructing the full information (i.e. intensity and phase) of the light from an object^1^. Since its invention, holographic-related technologies have been widely applied in numerous areas such as optical display, imaging, data storage, encryption and metrology^2^. Holography based on conventional optical devices such as spatial light modulator (SLM) suffers from the disadvantages of low resolution and small field-of-view, hindering its practical applications. In contrast, one can access the high resolution, ultra-thin thickness, and high-performance counterpart by replacing the conventional components with metasurfaces^3–7^. Metasurfaces, composed of two-dimensional subwavelength arrays of nanoscale scatters, allow for manipulating lights with mutiple degree of freedoms (DoFs), providing a new generation of versatile platforms for optical multiplexing holography^6,8,9^. In this context, different physical dimensions of light such as wavelength^10–14^, incidence angle^15–17^, state of polarization (SoP)^18–26^ and time^27,28^, have been exploited as independent information channels for holographic systems. Having almost exhausted the existing physical dimensions for multiplexing holography, the angular momentum (AM) dimension of light has emerged as a new opportunity.

The optical AM, a quantum mechanical description of the photon, has boosted numerous applications in both classical and quantum optical fields, including optical tweezers^29^, the spin-Hall effect^30,31^, quantum microscopy^32^, and so forth. The AM is categorized as orbital angular momentum (OAM) and spin angular momentum (SAM). The former is associated with a helical phase front exp(*ilφ*) and owns unbounded values denoted by *lℏ* (*l* is the topological charge, *φ* is the azimuthal angle, and *ℏ*, reduced Planck’s constant, is the quantization of AM). The latter, often manifested as a circular polarization that is associated with the direction of the electric field oscillating in time, owns a limited quantized value of ± ℏ per photon^31,33^. By means of appropriate spatial frequency sampling of a hologram in momentum space, OAM has been implemented as an independent information carrier for optical holography^34–37^. Owing to the unbounded helical mode and intrinsic orthogonality, OAM-multiplexed holography shows unprecedented capacity for optical information processing^38^. Moreover, the linearly polarization channels are added to the OAM-dependent holography, with which the information capacity is further enhanced^36^. On the other hand, the SAM, an equivalent physical dimension of OAM, has also been explored for multiplexing holography, ranging from the spin-dependent to spin-decoupled ones^18,19,39^. Moreover, numerous efforts have been devoted to generating the full-polarization vectorial holography that originates from the superposition of the two SAM eigenstates in the output field^17,20,26,40–42^. The full-polarization vectorial holography can offer unlimited multiplexing channels in principle due to the capacity of controlling the arbitrary polarization vectors on the Poincare sphere (PS), showing great potential for high-capacity optical encryption. Despite these thrilling achievements, adding the SAM dimension to the existing OAM-multiplexed holography and spatially manipulating the full-polarization vectors simultaneously, has not yet been investigated. To achieve this multi-functionality meta-hologram, the most intuitive design approach is segmenting or interleaving several kinds of meta-atoms, each of which corresponds to a specific functionality^26,43,44^. Such methods will set a restriction on the efficiency (reciprocal value of the functionality number) and introduce undesirable cross-talks^43,45^. To overcome these drawbacks, a metasurface consisting of non-interleaved meta-atoms would be an excellent choice^11,21,46,47^. However, up to now, the realization of AM holography by using either the interleaved or the non-interleaved strategies, has remained elusive. The intrinsic reason is the lack of a feasible physical mechanism to fully regulate the two OAM and SAM eigenstates in the output field.

Here, we theoretically and experimentally provide the AM-holography paradigm based on fully synergy of the SAM and OAM via a minimalist metasurface. The design methodology of AM-holography depends on independently controlling the two spin eigenstates and arbitrary overlaying them in each operation channel, thereby spatially modulating the resulting waveform at will. For the proof of the concept, we demonstrate an AM meta-hologram that can reconstruct two sets of distinct holographic images, namely, the spin-orbital locked (SOL) and spin-superimposed (SS) ones, resulting in the multi-dimensional and multi-channel holography determined by the incident AM. The multi-channel AM meta-holograms have offered additional security locks, allowing us to construct the advanced optical nested encryption platform for revolutionizing the existing optical encryption schemes suffered from either limited data capacity or low security. In the optical nested encryption strategy, the SOL and SS holographic images are employed to encrypt and decrypt the optical information in a specific sequence, making the encoded information invulnerable to certain brute-force attacks. Moreover, the nested encryption scheme theoretically owns unbounded information channels originating from the AM holography, catering to the ever-growing requirement of parallel high-security information transmission. It is worth noting that the design strategy is generalized and can be extended to achieve other waveform shaping functionalities such as spatial structured-light generation and polarization knots.

## Results

### Working principle and metasurface design

**Fig. 1:**
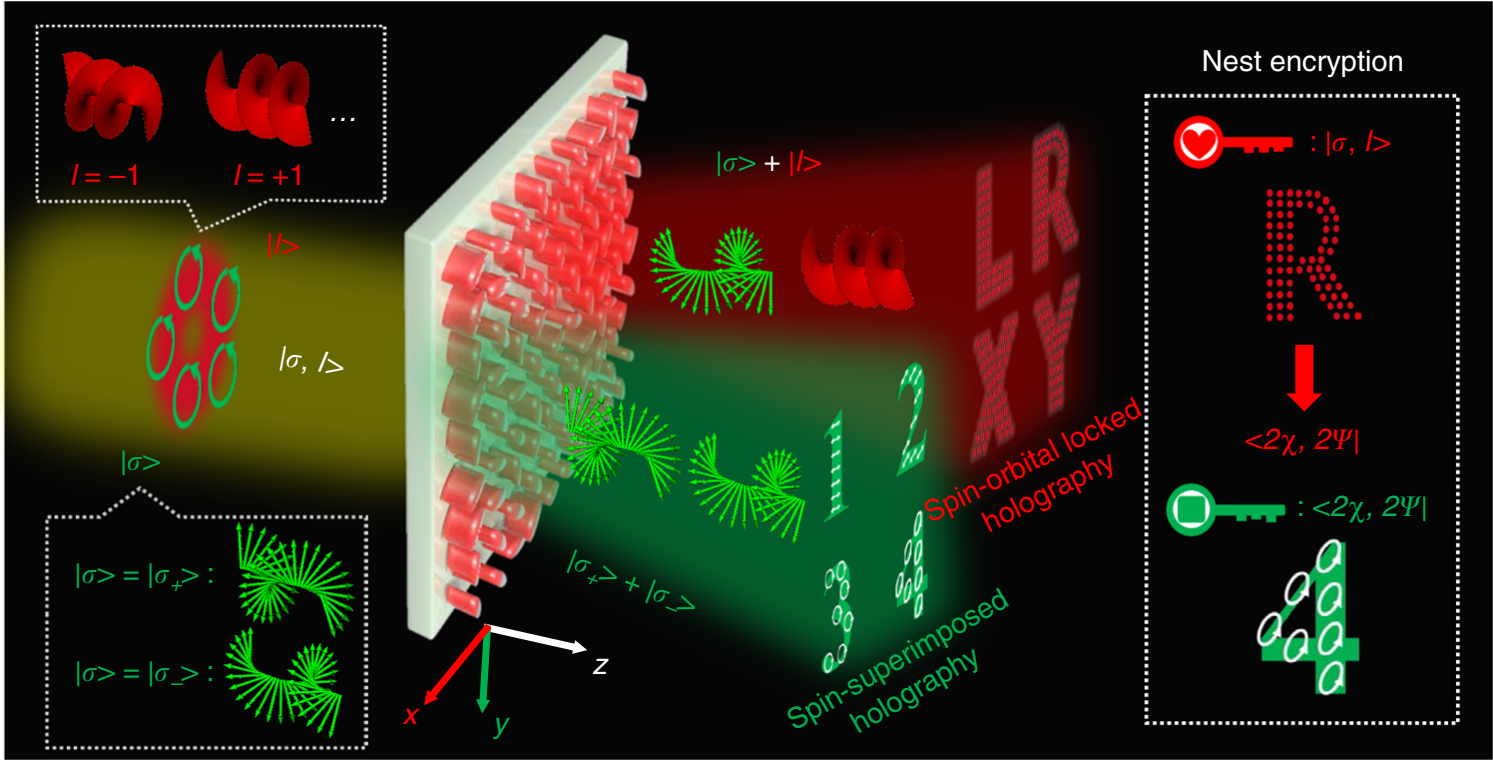
Schematic illustration of the AM holography for optical nested encryption. The AM holography depends on arbitrary superimpose the SAM and OAM eigenstates in the output field. For the spin-orbital locked holography (SOLH), the reconstruction of the four holographic images “L, R, X, Y” depends on the incident light carrying certain SAM and OAM values (indicated as |*σ*, *l* >). For the spin-superimposed holography (SSH), the four Arabic numbers “1, 2, 3, 4” with specific spatially distributed SoPs are reconstructed under the incident XLP Guassian light (indicated as |0, 0 >). For the optical nested encryption, the reconstructed SOL holographic images are used to perform as the keys (translated to certain SoPs represented as <2*χ*, 2*ψ* | , where *χ* and *ψ* indicate the ellipticity angle and azimuth angle, respectively.) of the next decryption and the encrypted information is fully unlocked by the generated SS holographic images

Figure 1 schematically illustrates the AM holography via a non-interleaved metasurface, which is capable of producing a series of AM-dependent holographic channels. Relying on the way that the SAM and OAM eigenstates are superimposed in the output field, we classify the AM holography as the spin-orbital locked holography (SOLH) and spin-superimposed holography (SSH). The superposition of the SAM and OAM states leads to the SOLH, which adds a new dimension, that is, the SAM, to the existing OAM-multiplexed holography^34,35,38^. Due to their mutual orthogonality, the SAM and OAM can be randomly combined to act as independent information carriers of the SOLH, leading to exponentially enhanced multiplexing channels compared to the OAM-multiplexed holography. Four target images of capital letters “L, R, X, Y” can be accurately reconstructed, where each pixel appears as a Gaussian spot, only when an incident beam carrying specific SAM and OAM eigenstates (represents as |*σ*, *l* >, where *σ* and *l* represent the values of SAM and OAM) illuminating on the meta-hologram. The arbitrary superposition of the two SAM eigenstates in each operation channel leads to the SSH, referred to as vectorial holography, which is capable of spatially controlling the polarization vectors. Here we define it as the SSH since such a definition is essentially more consistent with the design principle. For the SSH, four target images of Arabic numbers “1, 2, 3, 4” with specific spatially distributed state of polarizations (SoPs) are reconstructed under the *x*-linearly polarized (XLP) Gaussian incident light (represents as |0, 0 > ). Most importantly, the two kinds of holography operating at intrinsic orthogonal AM beams (with the Gaussian beam being treated as a special vortex beam with topological charge *l* = 0) result in independent holographic channels. Here we schematically demonstrate eight information channels to show the design principle, and the design strategy can be extended to obtain more independent holographic channels. For the optical nested encryption, the SOL holographic images are decrypted to provide the keys of the next decryption and the encrypted messages are fully unlocked by the generated SS holographic images (see details in the following discussion).

For the AM holography, the design principle relies on the superposition of the SAM and OAM states. As we know, the OAM property of incident beam can be addressed in the holographic images by appropriate spatial frequency sampling of a hologram in momentum space^35^. Hence, to achieve the AM holography via a monolithic non-interleaved meatsurface, the first key issue is the design of meta-toms capable of independently controlling the two output spin eigenstates (with the Jones vectors: |σ_+_>=[1,i]^*T*^ and |σ->=[1,-i]^*T*^). For this, the required metasurface is able to impart two independent phase profiles *φ*_*L*_(*x,y*) and *φ*_*R*_(*x,y*) on the two output spin eigenstates, respectively. Therefore, the light and metasurface interaction effect can be expressed as

1 $$J\left( {x,y} \right)\left| {\sigma _ - > = e^{i\varphi _L\left( {x,y} \right)}} \right|\sigma _ + >\, J\left( {x,y} \right)\left| {\sigma _ + > = e^{i\varphi _R\left( {x,y} \right)}} \right|\sigma _ - >$$

where *J*(*x,y*) represents the Jones matrix of the metasurface. *x* and *y* represent the coordinates of meta-atoms in the metasurface plane.

**Fig. 2:**
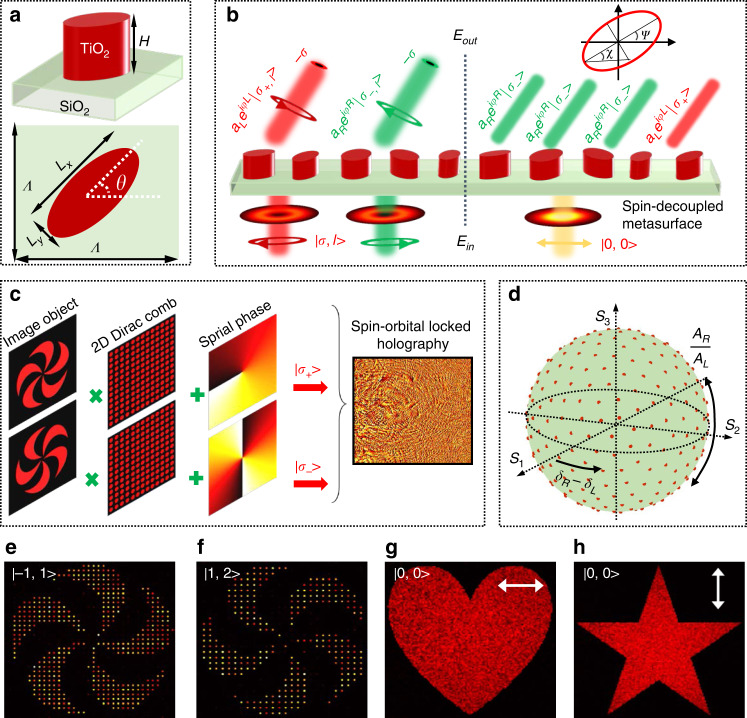
Principle and demonstration of the metasurface AM holography. **a** Top and side views of the meta-atom, in which the one can get the detailed parameters. **b** The principle of the non-interleaved spin-decoupled metasurface for AM holography, where each operation channel depends on arbitrary superimposing the two spin eigenstates. **c** The design strategy of the SOLH meta-hologram. **d** Schematic of the reconstructed SoPs on the entire PS with phase difference *δ*_*R*_-*δ*_*L*_ ranging from 0 to 2π and amplitude *A*_*R*_ and *A*_*L*_ ranging from 0 to 1. **e**–**h** Numerical reconstruction of the four distinctive holographic images through a designed four-channel AM meta-hologram. The white arrows represent the extracted SoPs

Figure 2a shows the side and top views of a designed meta-atom, which consists of a TiO_2_ elliptical nanoblock placed on a silica substrate. The detailed parameters are the height *H* = 1 μm, subwavelength lattice constant *Λ* = 0.45 μm, and in-plane dimensions (major axis *L*_*x*_, minor axis *L*_*y*_ and the orientation angle *θ*). For the meta-atoms with birefringence, the Jones matrix can be described^19,48^ as:

2 $$J\left( {x,y} \right) = R\left( { - \theta \left( {x,y} \right)} \right)\left[\begin{array}{*{20}{c}} {e^{i\delta _x\left( {x,y} \right)}} & 0 \\ 0 & {e^{i\delta _y\left( {x,y} \right)}} \end{array}\right]R(\theta \left( {x,y} \right))$$

where *δ*_*x*_(*x,y*) and *δ*_*y*_(*x,y*) represent the spatial propagation phase shifts along the two symmetry axes of the meta-atom at each coordinate (*x,y*). *R* is a 2×2 rotation matrix. *θ*(*x,y*) represents the orientation angle of the meta-atom, which determines the geometric phase shift.

From Eqs. (1) and (2), metasurface with spin-decoupled phase control can be derived as (see section 1 of the Supporting Information for details):

3 $$\varphi _R\left( {x,y} \right) = \delta _x\left( {x,y} \right) - 2\theta (x,y)$$

4 $$\varphi _L\left( {x,y} \right) = \delta _y\left( {x,y} \right) + 2\theta \left( {x,y} \right) - \pi$$

5 $$\theta \left( {x,y} \right) = \left[ {\varphi _R\left( {x,y} \right) - \varphi _L\left( {x,y} \right)} \right]/4$$

Using Eqs. (3–5), one can arbitrarily and independently manipulate the phase shifts of the two spin eigenstates by proper designing the meta-atoms, which can provide the required three values (*δ*_x_, *δ*_y_ and *θ*) at any coordinate (*x,y*). The full-wave finite-difference time-domain simulations are performed to optimize the meta-atoms at a working wavelength of 635 nm (see section 2 of Supporting Information). Therefore, the in-plane dimensions and orientation angle of the meta-atom at each point of the final meta-hologram can be determined.

Then, the two spin eigenstates are arbitrary superimposed in each working channel, in which the OAM eigenstates are superimposed by the adoption of spiral phase profiles.

As a result, the AM holography that exhibits two kinds of holographic images is achieved via a single non-interleaved metasurface. Figure 2b illustrates the principle of the non-interleaved spin-decoupled metasurface for AM holography. When an arbitrary polarized light (which can be decomposed into the superposition of the two spin eigenstates) illuminates on the metasurface, independent phase shifts (*φ*_*R*_ and *φ*_*L*_) can be imparted on the two output spin eigenstates. Noting that, the output spin eigenstates own identical amplitude, which is treated as unity (*a*_*R*_ = *a*_*L*_ = 1) for simplicity in the following derivation (see section 3 of the Supporting Information for details). For the SOLH, the output components are spin flipped, and endowed with OAM-dependent phase profiles, which can be expressed as

6 $$\begin{array}{l}J\left( {x,y} \right)| {\sigma _{ + ,l} > = e^{i\varphi _R\left( {x,y} \right)}} |\sigma _{ - ,l} > J\left( {x,y} \right)| \sigma _{ - ,l} > \\\qquad\qquad\qquad\, = e^{i\varphi _L\left( {x,y} \right)} |\sigma _{ + ,l} >\end{array}$$

where *|σ*_*+*,*l*_ > = e^*ilδ*^ | *σ*_+_ > and *|σ*_-,*l*_ > = e^*ilδ*^ | *σ*_-_ > represent the incident circularly polarized (CP) vortex beam, *δ* = arctan(*y/x*) is the in-plane azimuth angle and *l* is the topological charge of the incident vortex beam. The physical mechanism and implement process of the SOLH is illustrated in Fig. 2c. Multiplying an image object with the OAM-dependent 2D Dirac comb function (with constant periodicity *g*) in the spatial frequency domain leads to an OAM-conserved holography. The sampling constant are determined by the spatial frequency of different spiral phase plates, which, in the paraxial limit, exhibit as different doughnut-shaped intensity distributions in the image plane based on the Fourier transform (see Fig. S3 in the Supporting Information). Superposing a spiral phase profile (with topological charge -*l*) on the OAM-conserved hologram and endowing them to a specific SAM state ultimately leads to the SOLH (see section 4 in the Supporting Information for details). As a result, SOL holographic images appear as Gaussian spots in each pixel only when an incident light that carry certain AM value of |*σ*, *l* > illuminating on the meta-hologram. The phase profile of each holographic image can be calculated by the modified Gerchberg-Saxton (G-S) algorithm (see Fig. S6 in the Supporting Information). Then, according to the harmonic strategy, the calculated holographic phase profiles are superposed and ultimately leads to an SOLH. For the SSH, the output SoP at an arbitrary spatial position is the superposition of the two spin eigenstates, as shown in the right panel of Fig. 2b. Here, the four outgoing components represent the specific case of generating an elliptical polarized channel, which composed of three *|σ*_-_ > and one *|σ*_*+*_> eigenstates, and the output SoP is represented by the polarization ellipse. For the general case, the output SoP (represented as *|S*>) of an arbitrary holographic channel is described as

7 $$\left| {S > = A_Re^{i\left( {\varphi (x,y) + \delta _R} \right)}} \right|\sigma _ - > + A_Le^{i(\varphi (x,y) + \delta _L)}|\sigma _ + >$$

where *φ*_*R*_(*x,y*) = *φ*(*x,y*) + *δ*_*R*_ and *φ*_*L*_(*x,y*) = *φ*(*x,y*) + *δ*_*L*_ represent the phase profiles imparted on the two output spin eigenstates, in which *φ*(*x,y*) represents the phase profile of the hologram. *A*_*R*_, *A*_*L*_ and *δ*_*R*_, *δ*_*L*_ are the amplitude and endowed phase shifts of the output RCP and LCP eigenstates, respectively. Therefore, the azimuth angle *ψ* and ellipticity angle *χ* can be expressed as

8 $$2\psi = \delta _R - \delta _L,2\chi = arcsin\frac{{A_R^2 - A_L^2}}{{A_R^2 + A_L^2}}$$

The entire azimuth angle 2*ψ* on the PS can be realized by changing the phase difference *δ*_*R*_-*δ*_*L*_ from 0 to 2π. The ellipticity 2*χ* covering the whole PS can be realized by changing the amplitude from 0 to 1. As shown in Fig. 2d, an arbitrary SoP that covers the entire PS can be generated by intentionally selecting the phase and amplitude of the two output spin eigenstates.

### Demonstration of the AM holography

To verify the design principle, we numerically design an AM meta-hologram with four holographic channels. Figure 2e–h shows the numerical calculated intensity profiles of the reconstructed holographic images (see section 5 of Supporting Information for details). Two images that own different helicities are chose as target images of the SOLH. As expected, the two images are well reconstructed with incident light that respectively carries certain AM value of |*σ* = −1, *l* = 1> and |*σ* = 1, *l* = 2> illuminating on the meta-hologram, as depicted in Fig. 2e, f. The two holographic images appear as Gaussian spots in each pixel, with the exhibited helicities in accordance with the SAM values of incident lights. For the SSH, we chose a heart-shape and a pentagram patterns as the objective images, which are assigned with two distinct SoPs represented as <2*χ* = 0*,2ψ* = 0| and <2*χ* = 0*,2ψ* = π | , respectively. The reconstructed holographic images can be achieved by switching the incident light to a XLP Gaussian beam and extracting a certain SoP via utilizing a specific analyzer. As depicted in Fig. 2g, h, the vectorial holographic images of the heart-shaped and pentagram patterns are well reconstructed, with the orthogonal SoP totally blocked. Therefore, we have demonstrated an AM meta-hologram with four independent holographic channels, which holds great potential applications in multi-channel display and optical anti-counterfeiting.

To further validate the design principle, we numerically design and experimentally demonstrate an AM meta-hologram with up to 16 independent channels. Eight capital letters “X, D, L, E, Y, A, R, F” are set as target images of the SOLH, each of which can be accurately reconstructed with CP light carrying specific |*σ*, *l* > . The corresponding SAM and OAM values of the eight capital letters “X, D, L, E, Y, A, R, F” are represented as | +1, −2 > , |+1, −1 > , |+1, +1 > , |+1, +2 > , |−1, −2 > , |−1, −1 > , |−1, +1> and | −1, +2 > , respectively. The objective images of the SSH are chose as eight Arabic numbers ranging from 1 to 8, which are assigned with eight distinct SoPs represented as <2*χ*, 2*ψ* | . The corresponding SoPs of the eight images are <0, 0 | , <0, π | , <0, π/3 | , <0, 4π/3 | , <0, 2π/3 | , <0, 5π/3 | , <arcsin(4/5), 0 | , and < -arcsin(4/5), π | , respectively. Subsequently, the phase profiles of the meta-hologram with sixteen channels are calculated by using the modified Gerchberg-Saxton (GS) algorithm, and endowed to the spin-decoupled metasurface pixel by pixel (see section 6 in the Supporting Information).

**Fig. 3:**
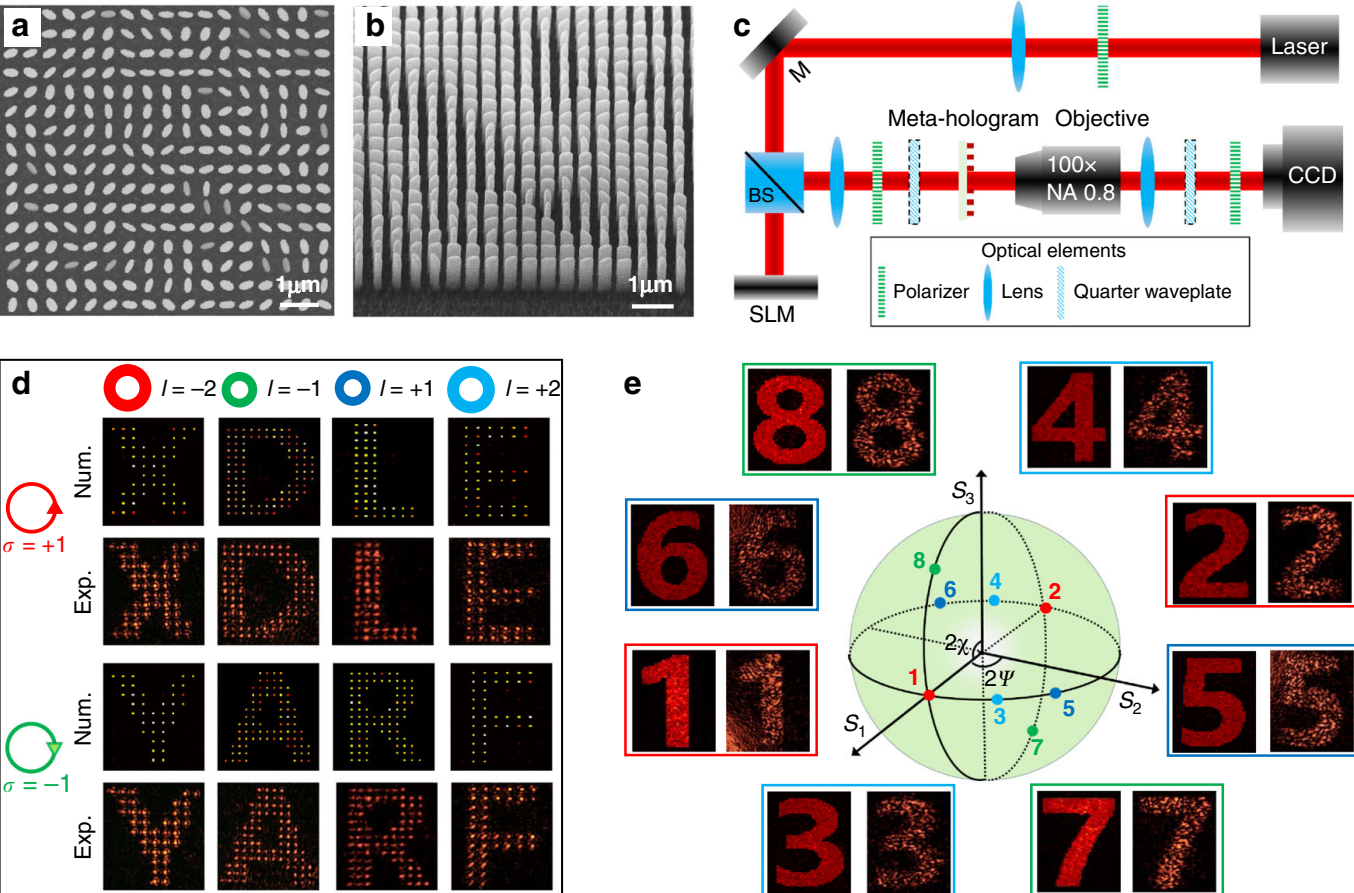
Experimental characterization of an AM meta-hologram with 16-bit independent holographic images. **a**, **b** Top and side views SEM images of the fabricated meta-hologram, with the scale bar to be 1 μm. **c** Characterizing system for the measurement of fabricated meta-hologram, where a spatial light modulator (SLM) along with a quarter waveplate (QWP) are used to generate required CP vortex beams. BS: beam splitter, SLM: spatial light modulator, M: mirror, CCD: charge-coupled device. The dashed boxes indicate that the QWP is removable in the certain measuring process. **d** Numerical and experimental reconstruction of the eight distinctive SOL holographic images through incident CP vortex beams with specific |*σ*, *l* > . **e** Numerical and experimental reconstruction of the eight distinctive SS holographic images through a XLP incident light and extracting certain SoPs via utilizing specific analyzers. The corresponding SoPs of the eight holographic images indicate by eight points on the PS

Then, we fabricate the meta-hologram sample by the standard electron beam lithography (EBL) and atomic layer deposition (ALD) followed by etching technique. The fabricated meta-hologram sample has a total of 480 × 480 pixels and with a footprint of 216 μm × 216 μm. The top and side views of the scanning electron microscopy (SEM) images of the fabricated sample are shown in Fig. 3a, b, respectively. Then, we characterize the AM meta-hologram by using an experimental set-up, as shown in Fig. 3c. A spatial light modulator (SLM) along with a quarter waveplate (QWP) are adopted to generate the required CP vortex beams. The QWP and linear polarizer pair are placed in front of the CCD, aiming at blocking the undesirable polarization vector. Figure 3d shows the numerically and experimentally reconstructed eight distinctive SOL holographic images. The eight target images can be well reconstructed with incident light carrying correct |*σ,l* > , otherwise the meta-hologram generating a series of misleading holographic images (see section 7 in the Supporting Information). Then, we characterize the spin-superimposed holographic images of the designed meta-hologram. For creating a XLP Gaussian incident light |0,0 > , the QWP between the SLM and the meta-hologram is removed. Figure 3e shows the numerically and experimentally reconstructed eight SS holographic images, with the SoPs are indicated on the PS. Here every two of the eight SS holograhic images (assigned with orthogonal SoPs) are elaborately set to superposition with each other, resulting in four superposition images. As expected, the image with a certain encoded SoP can be extracted without any cross-talk, and the other images will coexist with inevitable cross-talk (see section 7 in the Supporting Information). As a result, eight SS holographic images of Arabic numbers from 1 to 8 can be well reconstructed by extracting the specific SoPs: <0,0 | , <0,π | , <0,π/3 | , <0,4π/3 | , <0,2π/3 | , <0,5π/3 | , <arcsin(4/5),0 | , and < -arcsin(4/5),π | , respectively. For the designed AM meta-hologram with 16-bit independent channels, the performance of the reconstructed holographic images is not as good as that with only several operating channels. It can be observed that the experimental results show well agreement with the numerical ones. The discrepancy between the two results can be attributed to the fabrication imperfection including etching dose, roughness of surface, and deformation of shapes and the experimental measurement error.

### AM meta-hologram for optical nested encryption

**Fig. 4:**
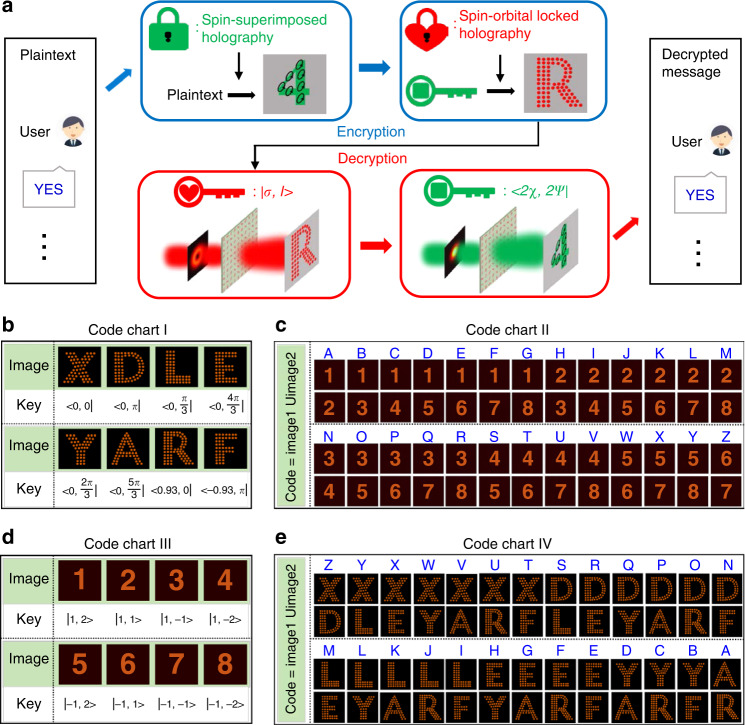
Schematic illustration of the optical nested encryption and demonstration of the code charts. **a** Conceptual illustration of the optical nested encryption scheme based on the AM meta-hologram. The optical information is successively encrypted by the two kinds of independent holography, which are represented by a red lock and a green lock, respectively. **b**, **d** Demonstration of code chart I and III, with the SOL and SS holographic images are translated to constitute keys for next decryption, respectively. **c**, **e** Demonstration of code chart II and IV, where the alphabet from A to Z are encoded. The coding rule is that each letter corresponds to a union set of two holographic images

The advent of metasurfaces has unlocked a new generation of platform for optical encryption with high capacity and security by fully exploiting the abundant DoFs of light. However, the simple information encrypted strategy—directly using a single DoF of light or the combination of them to be the encryption keys, making the encrypted data vulnerable to certain brute-force attacking algorithms^49–51^. For the designed AM meta-hologram, it is able to obtain two kinds of independent holographic images under different incident beams, which introduces additional DoF in security locks for optical encryption compared with the meta-hologram owns only one kind of holographic image. As a proof of concept, we demonstrate an optical nested encryption scheme for high-capacity optical information transfer with unprecedented level of security. Figure 4a shows the conceptual illustration of the optical nested encryption platform, where the SOL and SS holographic images are employed to encrypt and decrypt the optical information in a specific sequence. In the encryption mechanism, the optical information (termed as plaintext) is firstly encoded as SS holographic images, and then the corresponding keys are encrypted by the SOL holographic images. The plaintext information for different users is finally encoded into a single AM meta-hologram (the detailed phase realization process is shown in Fig. S6 in the Supporting Information). Once the AM meta-hologram is designed, the final phases (containing the OAM phases) are determined. In the decryption mechanism, the whole process is reverse. First, specific CP vortex beams |*σ, l* > are normally incident on the meta-hologram, with the reconstructed SOL holographic images to perform as the keys (translated to certain SoPs) of the next decryption. Then, by using a XLP Gaussian beam along with these keys, one can extract the correct SS holographic images (with specific SoPs represented as <2χ, 2*ψ* | ), which are translated by the predesigned coding chart to fully unlock the transmitted optical information. It worth noting that the sequence of the two holographic images is exchangeable in the optical nested encryption scheme, which will further increase the encryption security. Figure 4b–e show the four code charts using in the optical nested encryption scheme.

**Fig. 5:**
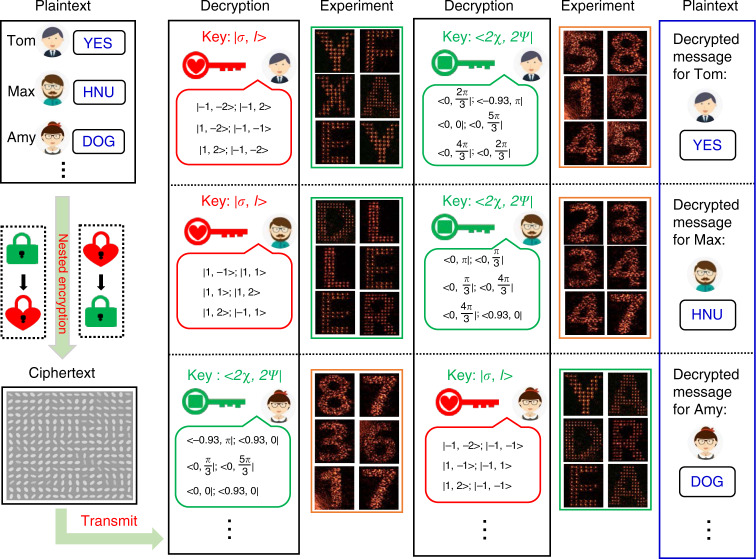
Experimental demonstration of using the designed 16-bit AM meta-hologram for optical nested encryption. The messages for Tom, Max, Amy, and so forth are all nested encrypted on one metasurface that termed as ciphertext. Identical samples are sent to multiple receivers, who can decrypt their respective messages according to the customized keys

With such an encryption scheme, multiple encoding data can transmit between multi users with high security, as shown in Fig. 5. Various plaintext messages (such as “YES”, “HUN”—the abbreviation of Hunan University and “DOG”) required to send to multiple users such as Tom, Max, Amy and so forth. These messages are nested encrypted by the two holographic images in a specific sequence and encoded into a single meta-hologram that termed as ciphertext. Then, identical meta-hologram samples along with the customized keys and code charts are sent to the users. Here, the messages for Tom and Max are encrypted with an identical sequence, and the message for Amy is encrypted with a distinct sequence. For Tom and Max, by using the received keys, they can obtain SOL holographic images consisting of capital letters, which are translated to the keys corresponding to second decryption via the predefined code chart I, as shown in Fig. 4b. With the second keys, the users are then able to implement to the second decryption—extracting the specific SS holographic images, that is the Arabic numbers from 1 to 8. Noting that, two arbitrary reconstructed SS holographic images are combined to represent the capital letters from “A” to “Z” (as shown in Fig. 4c), which does not directly expose the encrypted information and increase the security. Consequently, Tom and Max read out the information “58; 16; 45” and “23; 34; 47”, which can be respectively translated to the plaintext messages “YES” and “HUN” according to the code chart II in Fig. 4c. While Amy obtains the message via an entirely different decryption process. By using the received keys, she can obtain the Arabic numbers, which are translated to the keys for second decryption via the predefined code chart III, as shown in Fig. 4d. With the second keys, Amy is then able to implement to the second decryption. Consequently, Amy read out the information “YA; DR; EA”, which can be translated to the plaintext messages “DOG” according to the code chart IV (shown in Fig. 4e).

By using the proposed optical nested encryption platform, one can transmit multiple messages in parallel with ultra-high security. Anyone who want to access the information only when he or she possesses all four indispensable units including the metasurface sample (ciphertext), customized keys, two set of code charts and especially the specific decryption sequence. Crucially, the encryption sequence of two holographic algorithms is exchangeable, and the customizable code charts and character encoding system can be changed accordingly. In an extremely case that the physical device, transported keys and coding charts are all cracked by the thieves, they can only obtain a series of useless images (without knowing the specific decryption sequence). Therefore, it is almost impossible to access the information without the accurate one-by-one decryption process. Moreover, in principle, the demonstrated optical nested encryption scheme owns unlimited information channels, owing to the unbounded multiplexing channels of the SOLH and SSH.

## Discussion

Here, we designed and demonstrated the realization of the AM holography at a working wavelength of 635 nm. The design strategy is general and can be scaled to achieving the AM holography in other wavelength regions. In the main text, we have demonstrated the using of OAM states ranging from −2 to +2 (with the total number of OAM states is 4) for holographic multiplexing. For massive information transmission in practical applications, the higher order OAM states must be taken into consideration. Here, we numerically demonstrate the concept for using higher order OAM states. The OAM states with topological charges ranging from 1 to 12 are taken into consideration (with the total number of OAM states is larger than 10). For this, we design an AM meta-hologram with 32 independent channels (see section 8 in the Supporting Information for details). Although with reduced signal to noise ratio, the desired capital letter is well reconstructed (with each pixel appear as Gaussian spots) under incident beam with correct SAM and OAM values. Moreover, eight SS holographic images of Arabic numbers from 1 to 8 can also be well reconstructed (under a XLP Gaussian incident light) by extracting the specific SoPs.

It is worth noting that the multiplexing channels of the SOL holography and SS holography are both unbounded in principle. However, due to the phase-only modulation in AM holography design, the final multiplexing channels of the AM holography are still limited. To solve this issue, one possible way is the adoption of complex-amplitude modulation, with which the OAM multiplexing channels of up to 200 have been demonstrated^35^. Recently, using the interference effect among multi meta-atoms, the spin-decoupled complex-amplitude modulation has been demonstrated for near-field nanoprinting and far-field holography^44^. The spin-decoupled complex-amplitude modulation scheme can be adopted to design the AM holography, with which the multiplexing channels can be further enhanced.

In conclusion, we have proposed and experimentally demonstrated the realization of an AM meta-hologram with up to 16 independent AM-dependent operation channels via a single-layer, non-interleaved metasurface. We have explored the underlying mechanism of the AM holography, which relies on independently controlling the two spin eigenstates and arbitrary overlaying them in the output field. By illuminating the meta-hologram with incident light carrying correct SAM and OAM, eight predesigned SOL holographic images are well reconstructed at the imaging plane. The other eight SS holographic images can be obtained by switching the incident light to a XLP Gaussian beam, and simultaneously extracting the specific SoPs of the output field. The AM meta-hologram shows exponentially increased multiplexing channel compared with the OAM-multiplexed one, and owns the capacity of achieving two kinds of independent holographic images, which would introduce additional security locks for high-capacity optical encryption. Furthermore, we experimentally demonstrate an optical nested encryption scheme for multi-user optical information transmission, which shows ultra-high data capacity and security and is invulnerable to certain brute-force attacks. It’s worth noting that our proposed design strategy is capable of spatially modulating the waveform at will, holding promising applications in vector beam generation, optical communication, virtual and augmented reality and beyond.

## Materials and methods

### Structure fabrication

The samples were fabricated using electron beam lithography (EBL) along with the etching technique. First, a 1000 nm-thick polymethyl methacrylate (PMMA) electron-beam resist layer was spin-coated at 2000 rpm on the transparent silica substrate with an ITO film layer and baked on a hot plate for 4 min at 180 °C. Next, the sample was exposed by EBL with a 100 KV voltage and a beam current of 200 pA. Subsequently, we put the exposed sample in a mixed solution of isopropanol and methyl isobutyl ketone (IPA: MIBK = 3:1) for 1 min, and then fixed it in the IPA solution for 1 min at room temperature. Later, we used the atomic layer deposition (ALD) system to fill the exposed area with 220 nm TiO_2_. We use PMMA for the positive photoresist exposure process, which is void before deposition. Then the deposited thickness of TiO_2_ is related to the semi-minor axis of the maximum meta-atom. After this process, there will be a layer of 220 nm TiO_2_ on the top of the entire sample, and we removed it by ion beam etching (IBE) in the next process. After removing the TiO_2_ on the top layer, we used reactive ion etching (RIE) to remove the resist. Finally, the TiO_2_ nanostructures with a high aspect ratio (of up to 10) are obtained.

### Numerical simulations

Numerical simulations of the meta-holograms were carried out using commercial software Lumerical Solutions based on a finite difference time domain method. The period of meta-atoms was set as 450 nm. In simulation, perfectly matched layers were used for calculations of the meta-holograms. The substrate was included in the simulations. The refractive index of SiO_2_ was taken as 1.46 at the operating wavelength of 635 nm. The refractive index of the TiO_2_ was the measurement result by the ellipsometer.

## Supplementary information

Supplementary Information for Angular Momentum Holography via a Minimalist Metasurface for Optical Nested Encryption

## Data Availability

Data underlying the results presented in this paper are available from the corresponding author upon reasonable request.
