# Supplementary material for: Angular momentum holography via a minimalist metasurface for optical nested encryption
Source: Light Sci Appl. 2023 Mar 28;12:79. doi: 10.1038/s41377-023-01125-2 (PMC10050323; doi:10.1038/s41377-023-01125-2)
Supplement: Supplementary file 1 — Supplementary Information for Angular Momentum Holography via a Minimalist Metasurface for Optical Nested Encryption [file 41377_2023_1125_MOESM1_ESM.doc]

**Supplementary Information for**

**Angular Momentum Holography via a Minimalist Metasurface for Optical Nested Encryption**

Hui Yang^1,2^, Peng He^1^, Kai Ou^3^, Yueqiang Hu^1,4,*^, Yuting Jiang^1^, Xiangnian Ou^1^, Honghui Jia^1,4^, Zhenwei Xie^2,*^, Xiaocong Yuan^2^, and Huigao Duan^1,4,*^

^1^National Research Center for High-Efficiency Grinding, College of Mechanical and Vehicle Engineering, Hunan University, Changsha 410082, China

^2^Nanophotonics Research Center, Shenzhen Key Laboratory of Micro-scale Optical Information Technology, Institute of Microscale Optoelectronics, Shenzhen University, Shenzhen 518060, Guangdong, China

^3^Institute of Precision Optical Engineering, School of Physics Science and Engineering, Tongji University, Shanghai 200092, China

^4^Greater Bay Area Institute for Innovation, Hunan University, Guangzhou 511300, Guangdong Province, China

**Corresponding authors*: huyq@hnu.edu.cn; ayst3_1415926@sina.com; duanhg@hnu.edu.cn

**1. Metasurface designed for independent controlling the two spin eigenstates**

For meta-atoms with elliptical cross-sections that exhibit birefringence, they can be described by the Jones matrix [1, 2] as:

$\begin{matrix} J(x,y)=R(\text{-}\theta(x,y))[\begin{matrix} e^{i\delta_{x}(x,y)} & 0 \\ 0 & e^{i\delta_{y}(x,y)} \end{matrix}]R(\theta(x,y)) \\ =[\begin{matrix} \cos\theta(x,y) & \text{-}\sin\theta(x,y) \\ \sin\theta(x,y) & \cos\theta(x,y) \end{matrix}][\begin{matrix} e^{i\delta_{x}(x,y)} & 0 \\ 0 & e^{i\delta_{y}(x,y)} \end{matrix}][\begin{matrix} \cos\theta(x,y) & \sin\theta(x,y) \\ \text{-}\sin\theta(x,y) & \cos\theta(x,y) \end{matrix}] \end{matrix}$ (S1)

where *δ_x_*(*x,y*) and *δ_y_*(*x,y*) represent the propagation phase shifts under two orthogonal linearly polarized (LP) lights along the meta-atoms’ two symmetry axes. *x* and *y* represent the coordinates of meta-atoms in the metasurface plane. *θ*(*x,y*) represents the orientation angle of the meta-atom which determines the geometric phase shift and *R* is a 2×2 rotation matrix.

As we know, an arbitrary polarized light beam normally incident onto the metasurface can be decomposed into the two spin eigenstates (with the Jones vectors represented as |σ-> = [1,-i]*^T^* and |σ_+_> = [1,i]*^T^*), respectively. In order to realize two completely independent phase profiles on the two spin eigenstates, both the propagation and geometric phases are utilized. The spin-decoupled metasurface should be designed to endow two independent phase profiles *φ_R_*(*x,y*) and *φ_L_*(*x,y*) to the two output spin eigenstates, respectively. Hence, the spin-decoupled metasurface can be described by a Jones matrix *J*(*x,y*) which satisfies

$J(x,y)|\sigma_{+}\rangle=e^{i\phi_{R}(x,y)}|\sigma_{-}\rangle\begin{matrix} & J(x,y)|\sigma_{-}\rangle=e^{i\phi_{L}(x,y)}|\sigma_{+}\rangle\end{matrix}$ (S2)

After matrix inversion of the Eq. S2, we can calculate the Jones matrix as

$J(x,y)=\left[\begin{matrix} \frac{e^{i\phi_{R}(x,y)}+e^{i\phi_{L}(x,y)}}{2} & \frac{ie^{i\phi_{L}(x,y)}-ie^{i\phi_{R}(x,y)}}{2} \\ \frac{ie^{i\phi_{L}(x,y)}-ie^{i\phi_{R}(x,y)}}{2} & \frac{-e^{i\phi_{R}(x,y)}-e^{i\phi_{L}(x,y)}}{2} \end{matrix}\right]$ (S3)

Using Eq. S3, we can calculate the eigenvalues (*μ*_1_,*μ*_2_) and eigenvectors (ν_1_,ν_2_) as

$\mu_{1}=e^{i[(\phi_{R}(x,y)+\phi_{L}(x,y))/2]}\begin{matrix} & \end{matrix}\mu_{2}=e^{i[(\phi_{R}(x,y)+\phi_{L}(x,y))/2-\pi]}$ (S4)

$\nu_{1}=[\begin{matrix} \cos\frac{1}{4}[\phi_{R}(x,y)-\phi_{L}(x,y)] \\ \sin\frac{1}{4}[\phi_{R}(x,y)-\phi_{L}(x,y)] \end{matrix}]\begin{matrix} & \end{matrix}\nu_{2}=[\begin{matrix} -\sin\frac{1}{4}[\phi_{R}(x,y)-\phi_{L}(x,y)] \\ \cos\frac{1}{4}[\phi_{R}(x,y)-\phi_{L}(x,y)] \end{matrix}]$ (S5)

Using the symmetric and unitary conditions, the Jones matrix can be expressed as

(S6)

where *β* is a diagonal matrix and *U* is a real unitary matrix.

Compared Eqs. S1 with S6, spin-decouped phase control can be achieved and expressed as

$\phi_{R}(x,y)=\delta_{x}(x,y)-2\theta(x,y)$ (S7)

$\phi_{L}(x,y)=\delta_{y}(x,y)+2\theta(x,y)-\pi$ (S8)

$\theta(x,y)=[\phi_{R}(x,y)-\phi_{L}(x,y)]/4$ (S9)

From Eqs. S7-S9, one can arbitrarily and independently manipulate phase shifts of the two spin eigenstates by elaborately designing three parameters, that is *δ*_x_(*x,y*), *δ*_y_(*x,y*) and *θ*(*x,y*). These parameters can be further specific to the geometric parameters (*L_x_*, *L_y_* and *θ*) of the meta-atoms.

**2. The design of the meta-atoms**

In order to investigate the transmission properties of the meta-atom, full-wave simulations are performed by using the finite-difference time-domain (FDTD) method. Figures S1(A-D) demonstrate the transmission properties of the meta-atoms, in which the simulated phases and amplitudes of the meta-atoms as functions the major and minor axes of the meta-atom for the two linearly polarization (LP) incident lights. In these simulations, periodic boundary conditions are used along the *x*- and *y*-axes, and the perfectly-matched layer conditions are used along the *z* axis. It is observed that the required phase coverage of 0-2π can be obtained by varying the lateral dimension of the meta-atom, from a minimum to a maximum. Then, by using Eqs. 3-5 in the main text, one can arbitrarily and independently manipulate the phase shifts of the two spin eigenstates by proper designing the three parameters of the meta-atom (*L_x_*, *L_y_* and *θ*).


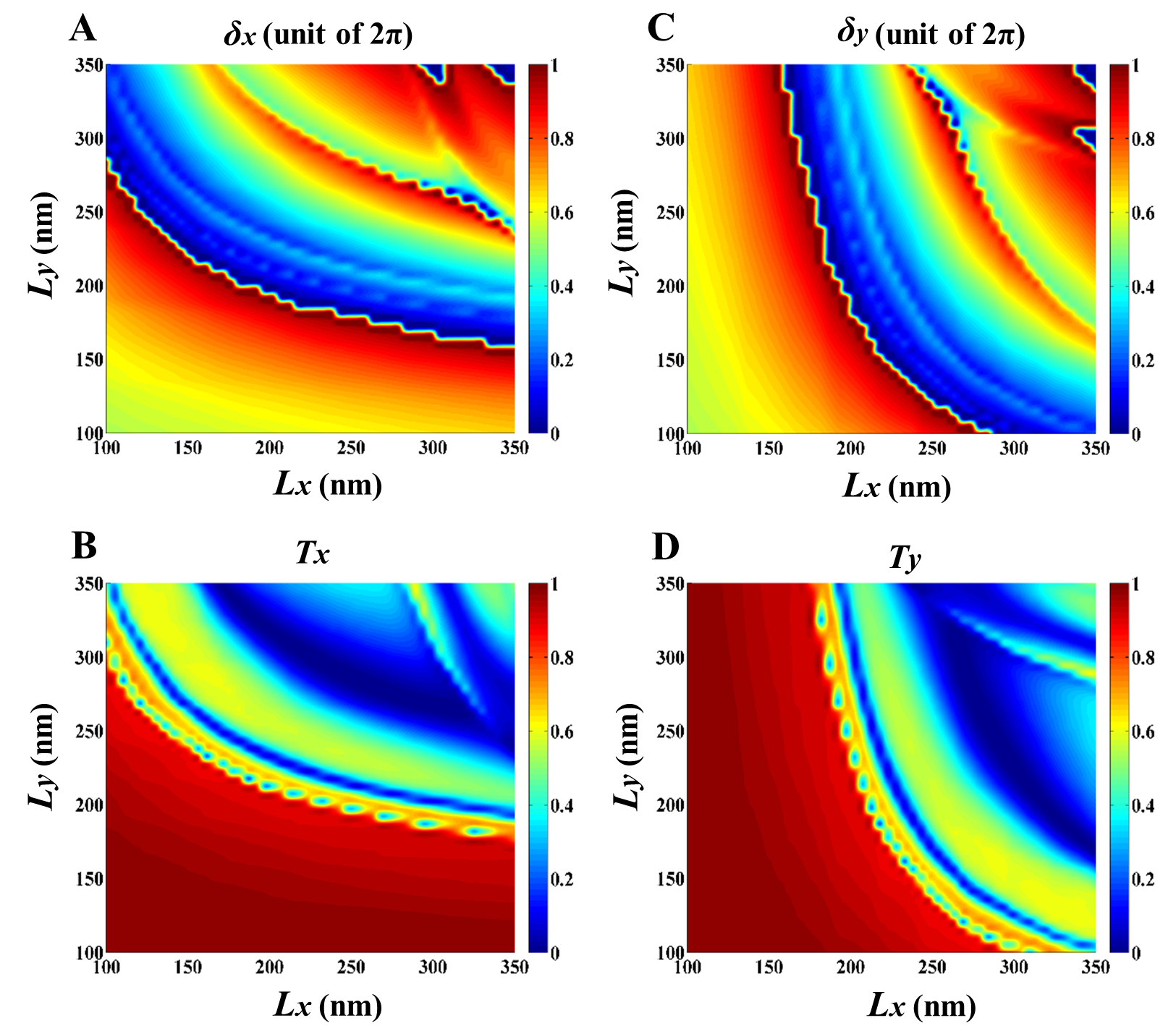


Fig. S1 (A) and (B) Phase shift and transmission as a function of meta-atom’s major and minor axes with *x*-linearly polarized (XLP) incident light. (C) and (D) Phase shift and transmission as a function of meta-atom’s major and minor axes with *y*-linearly polarized (YLP) incident light. In consideration of the practical manufacturing process, only elliptical nanoblocks with lengths (*L_x_* and *L_y_*) range from 100nm to 350nm are adopted.

**3. Spin-decoupled wavefront control with identical amplitude**

Figure S2 schematically illustrates a spin-decoupled metasurface (a single line metasurface is choose for simplify) for wavefront shaping under a XLP incident light. Due to the broken symmetry of the two spin states, the phase profiles produced by the two circularly polarized (CP) lights can be arbitrarily and independently endowed. As shown in Fig. S2, when illuminated with a LP incident light, which can be expressed as the combination of two CP beams (with identical amplitude). The metasurface can achieve two wavefront shaping functionalities, which can be expressed as

$|F_{R}\rangle=\sum_{j=1}^{M} P_{Rj}\exp(i\phi_{Rj})|\sigma_{-}\rangle\begin{matrix} & |F_{L}\rangle=\sum_{j=1}^{M} P_{Lj}\exp(i\phi_{Rj})|\sigma_{+}\rangle\end{matrix}$ (S10)

where |*F_R_*> and |*F_L_*> represent the two wavefront shaping functionalities. *M* is the number of the meta-atoms. *P_R_* and *P_L_* represent the polarization conversion efficiency of each meta-atom. For the case that the two wavefront shaping functionalities are identical, namely, the endowed phase shifts are identical and can be expressed as *φ_Rj_* = *φ_Lj_*. Noting that, the two polarization conversion efficiency for each meta-atom is identical, that is *P_Rj_* = *P_Lj_*. Therefore, Eq. S10 can be expressed as

$|F_{R}\rangle=\sum_{i=1}^{M} P_{Ri}\exp(j\phi_{Ri})|\sigma_{-}\rangle=\sum_{i=1}^{M} P_{Li}\exp(j\phi_{Li})|\sigma_{+}\rangle=|F_{L}\rangle$ (S11)

From Eq. S11, we can conclude that the two spin-decoupled wavefront shaping functionalities own identical amplitude. Here we just consider two functionalities, and this result remains valid for the spin-decoupled metasurface endowed with several wavefront shaping functionalities.

**
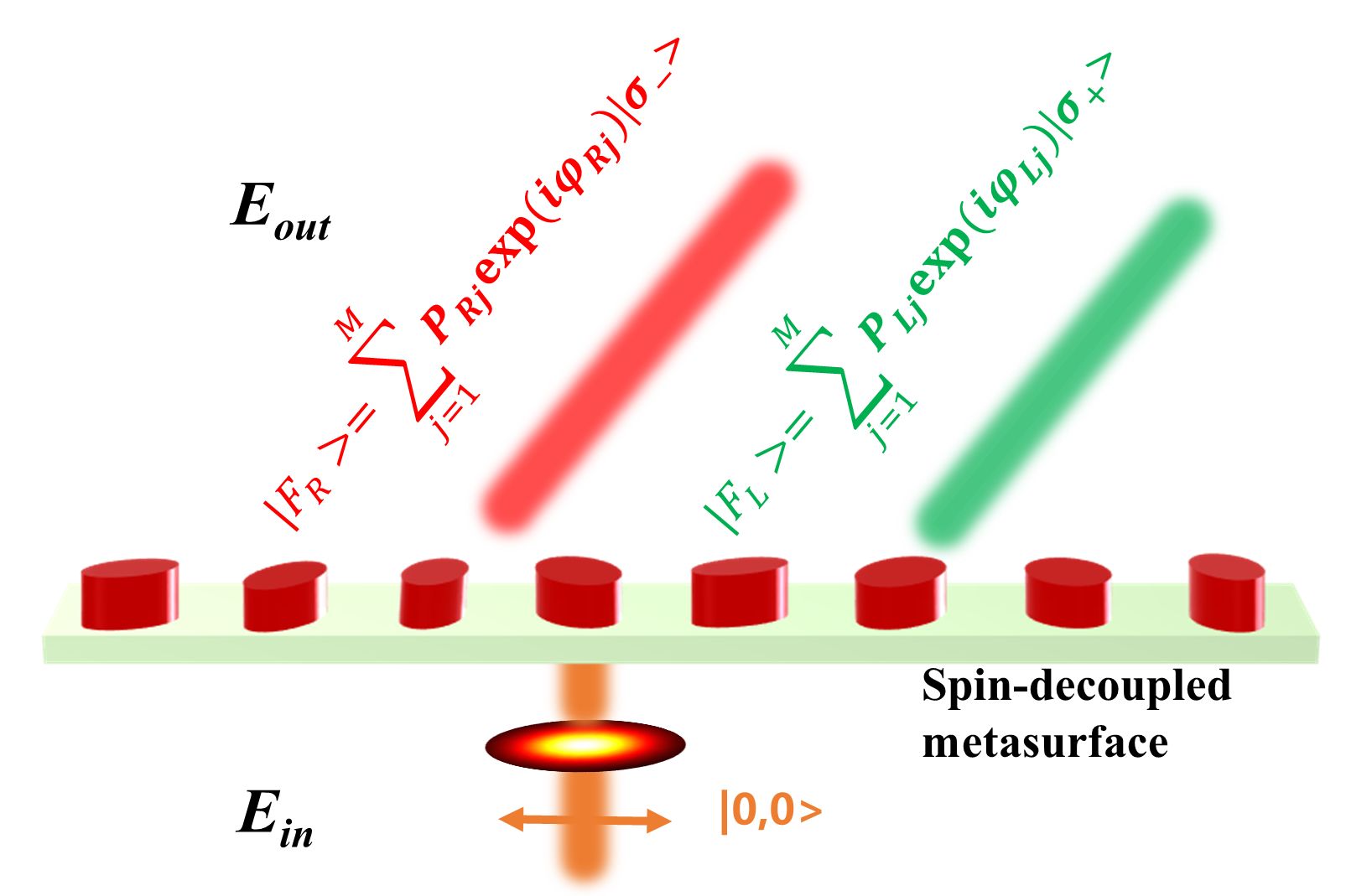
**

Fig. S2 Schematic illustration of a spin-decoupled metasurface for wavefront shaping under an incident XLP Guassian light represented as |0,0>. The spin-decoupled metasurface is able to achieve spin-decoupled wavefront shaping functionalities with identical amplitude.

**4. The design of the spin-orbital locked holography**

**4.1. The spatial frequency distribution of different incident OAM beams**

Considering the cylindrical symmetry of an optical beam, the Fourier transform of a complex helical wavefront can be calculated as [3]

$E(\rho,\theta)=FT(\exp(il\phi))=\frac{(-1)^{l+1}k}{f}\exp(il\phi)\int_{0}^{R} J_{l}(\frac{k}{f}r\rho)rdr$ （S13）

where FT represent the spatial Fourier transformation, *k*=2π/λ is the wavevector of incident light and *f* is the focal length of a Fourier lens. *r* and *ρ* are the radii in the hologram plane and image plane, respectively. *J_l_* is an *l*-th order Bessel function of the first kind.

For our experimentally fabricated meta-hologram with a physical size of 216μm by 216 μm and the subwavelength resolution (period of the meta-atom) is 450nm, resulting in the pixel number of 480 by 480. The reconstruction distance of meta-holograms set as 800 μm. Using Eq. S13, we calculated the spatial frequency distributions in the image plane of different incident OAM beams, as shown in Fig. S3. The spatial frequency distribution of a helical wavefront is represented by a doughnut-shaped intensity distribution in the image plane. The sampling constant of the meta-hologram in the image plane is determined as *g*.


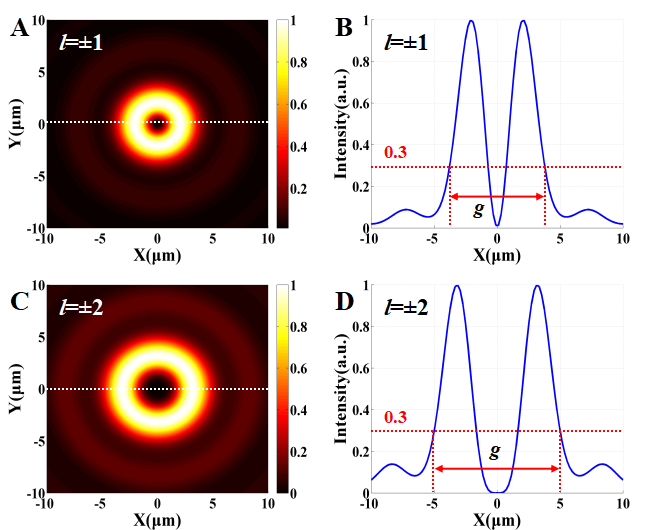


Fig. S3. Numerical calculation of the spatial frequency distributions in the image plane of different incident OAM beams. (A) The intensity distributions for incident OAM beams with topological charge value of *l* = ±1. (B) The corresponding intensity cross-section along *x*-axis. (C) The intensity distributions for incident OAM beams with topological charge value of *l* = ±2. (D) The corresponding intensity cross-section along *x*-axis. The red dotted lines labels out the positions used for defining the sampling constant *g*.

**4.2 Detailed design process of the spin-orbital locked holography**

For the spin-orbital locked holography (SOLH), the physical mechanism and implement process is illustrated in Fig. S4. An OAM-conserved hologram can be achieved via sampling the object image by a 2D Dirac comb function in the spatial frequency domain (with sampling constant *g*). The sampling constant determined by the spatial frequency of different spiral phase plates, which, in the paraxial limit, exhibit as different doughnut-shaped intensity distributions in the image plane based on the Fourier transform (see section 4.1 for details). By doing that, the OAM-information is preserved in the OAM-conserved holographic images. Then, a spiral phase plate with a phase distribution of -*lφ* is further added onto the OAM-conserved meta-hologram, resulting in an OAM-selective meta-hologram. Endowing the OAM-selective meta-hologram to a specific SAM state ultimately lead to the SOLH. As a result, holographic images appear as Gaussian spots in each pixel only when the CP vortex beams with specific SAM and OAM values |*σ,l*> illuminating on the meta-hologram. The phase profiles of the eight holographic images can be calculated by the modified Gerchberg-Saxton (G-S) algorithm, and superposed according to the harmonic resonance design strategy.


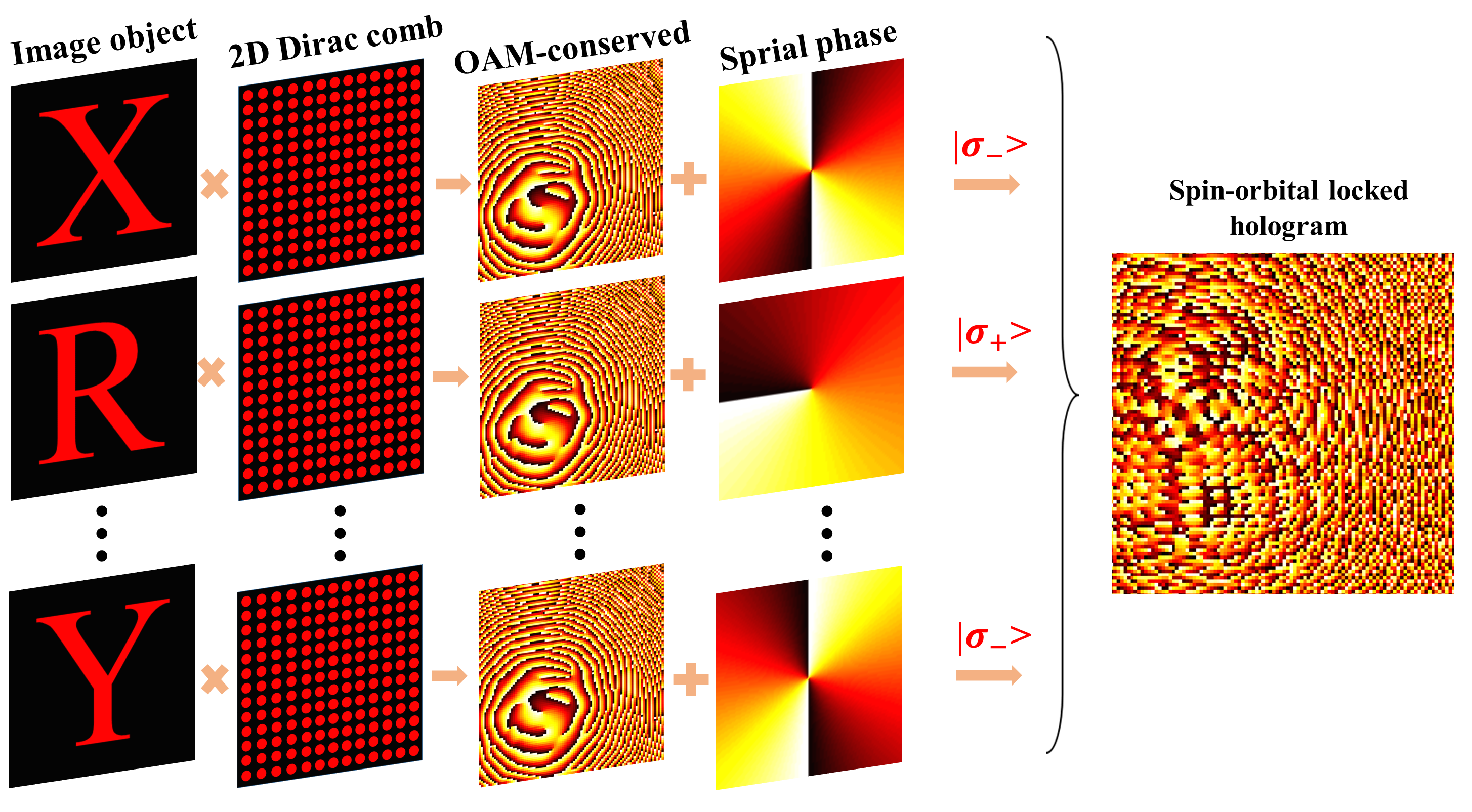


Figure S4. The design principle of the SOL meta-hologram. The implementation procedure is as followed. Multiplying an image object with the OAM-dependent 2D Dirac comb function (with constant periodicity *g*) in the image plane leads to an OAM-conserved hologram. Superposing a sprial phase profile (with topological charge -*l*) on the OAM-conserved hologram and endowing them to a specific SAM state ultimately lead to the SOL meta-hologram.

**5. Numerical demonstration of a four-channel AM meta-hologram**

To verify the design principle, we numerically design an AM meta-hologram with four holographic channels. Here two images that own different helicities are chose as target images of the SOLH, as shown in Figs. S5A and S5B. For the spin-superimposed holography (SSH), we chose a heart-shaped and a pentagram patterns as the objective images, which are assigned with two distinct SoPs represented as <2*χ* = 0*,2ψ* = 0| and <2*χ* = 0*,2ψ* = π|, respectively. Figures S5(E-H) shows the numerically calculated intensity profiles of the reconstructed holographic images. In the meta-hologram design, we set the two SOL holographic and SS holographic images are overlapped with each other. As expected, the two SOL holographic images are well reconstructed with incident light that respectively carries correct AM value of |*σ* = -1, *l* = 1> and |*σ* = 1, *l* = 2> illuminating on the meta-hologram, as depicted in Figs. S5E and S5F. The two holographic images appear as Gaussian spots in each pixel, with the exhibited helicities in accordance with the SAM values of incident lights. Such a characteristic can also provide an efficient way to resolving the optical singularity of incident light. The SS holographic images can be achieved by switching the incident light to a XLP Gaussian beam and extracting a certain SoP via utilizing a specific analyzer. As depicted in Figs. S5G and S5H, the SS holographic images of the heart-shaped and the pentagram patterns are well reconstructed, with the orthogonal SoP totally blocked. Therefore, we have demonstrated the AM holography via a single non-interleaved metasurface, which holds great potential applications in multi-channel display, optical anti-counterfeiting and optical communication.

We also demonstrate the case that the AM meta-hologram incident with light carrying AM values that are not the designed ones. The numerically calculated intensity profiles of the meta-hologram with incident light carrying AM value of |*σ* = 1, *l* = 1> and |*σ* = -1, *l* = 2> are shown in Fig. S5I and S5J, respectively. It can be observed that the resulting SOL holographic images appear as doughnut-shaped intensity distributions in each pixel (according to the OAM superposition principle). The two holographic images also exhibit helicities in accordance with the SAM values of the incident lights. Moreover, the numerically calculated intensity profiles of the meta-hologram under a XLP vortex incident light with topological charge of *l* = 1 and *l* = 2, are shown in Fig. S5K and S5L, respectively. The incident polarization direction and extracted SoP are represent by a red and a white double arrow line, respectively. It can be observed that the SS holographic images reconstruct well (with reduced intensity) by extracting the certain SoPs via utilizing specific analyzers. As we know, the XLP vortex beam can be decomposed as the superposition of two CP vortex beams. Therefore, for a specific incident XLP vortex beam, the SOL holographic images corresponding to the two spin eigenstates are both appear, as shown in Fig. S5K and S5L. The SOL holographic image appears as Gaussian spots in each pixel when the OAM values matching the designed ones. Otherwise, the SOL holographic image appears as doughnut-shaped intensity distributions in each pixel. From the above discussion, we can conclude that the SS holographic images can also be reconstructed via an incident XLP vortex beam. However, it simultaneously exposes the required SOL holographic image. Hence, to obtain two kinds of independent holographic images, the incident light should own different OAM values (the OAM value of a XLP beam is *l* = 0).

**
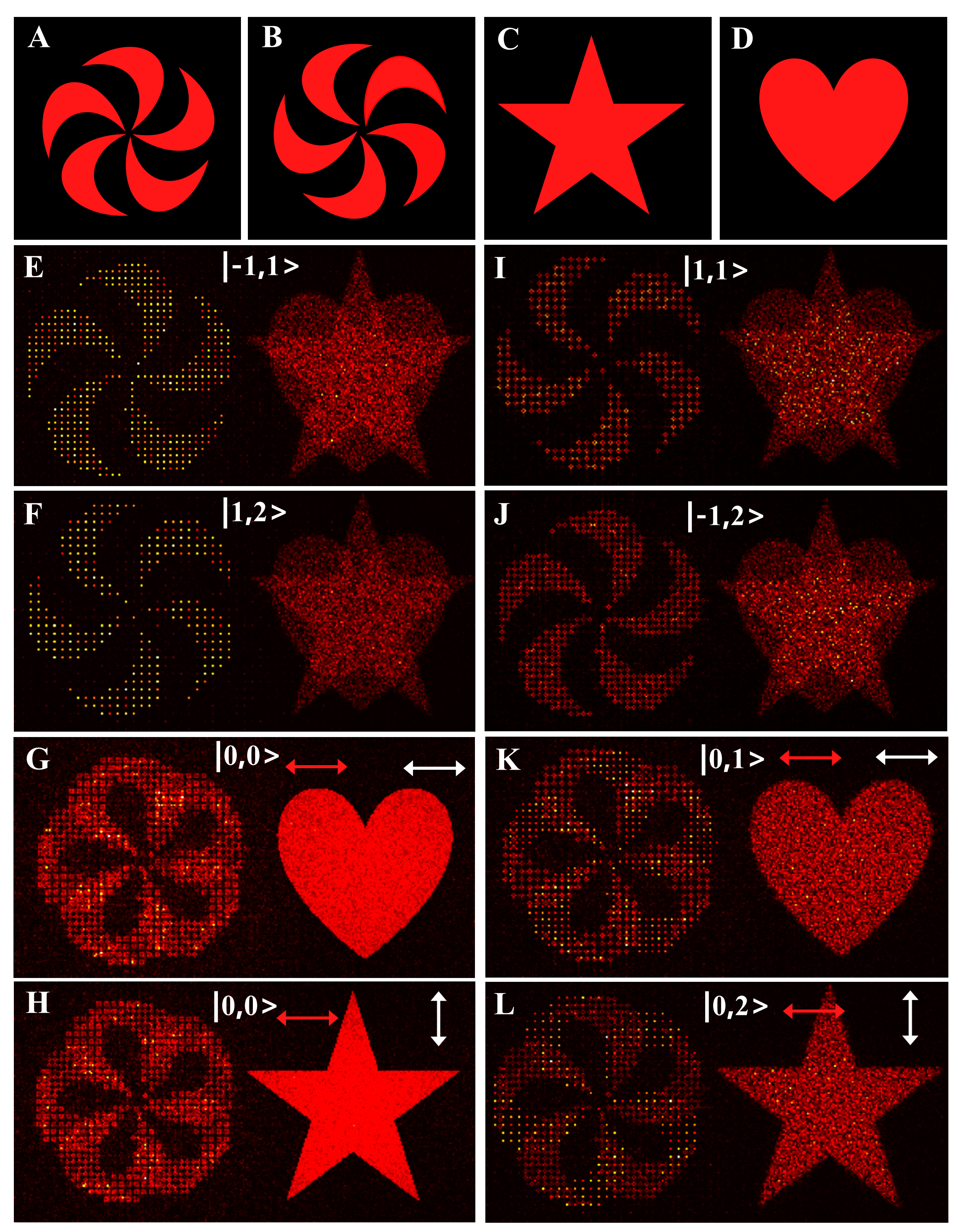
**

Fig. S5. (A) and (B) Target images of the SOLH, which are two five hakaze fans that own different helicities. (C) and (D) Target images of the SSH. (E) and (F) The numerically calculated intensity profiles of the meta-hologram with incident light that respectively carries correct AM value of |*σ* = -1, *l* = 1> and |*σ* = 1, *l* = 2> illuminating on it. (G) and (H) The numerically calculated intensity profiles of the meta-hologram under XLP incident light, and with a certain SoP is extracted via utilizing a specific analyzer. (I) and (J) The numerically calculated intensity profiles of the meta-hologram with incident light carrying AM value of |*σ* = 1, *l* = 1> and |*σ* = -1, *l* = 2>, respectively. (K) and (L) The numerically calculated intensity profiles of the meta-hologram under XLP vortex incident lights with topological charge of *l* = 1 and *l* = 2, and with a certain SoP is extracted via utilizing a specific analyzer. The incident polarization direction and extracted SoP are represent by a red and a white double arrow lines, respectively.

**6. The design of the 16 bits AM meta-hologram**

**6.1 Phase retrieval algorithm of the AM meta-hologram**

The flow chart of the phase retrieval process for the AM meta-hologram is depicted in Fig. S6. For the SoLH and SSH, the phase profiles of the eight objective holographic images can be calculated by using the OAM-selective GS algorithm and modified GS algorithm, respectively. Then, the corresponding phases profiles are multiplexed and endowed to the two spin eigenstates of the output beam. Finally, using a look up algorithm, the in-plane dimensions of the meta-atoms at each coordinate of the meta-hologram are determined.


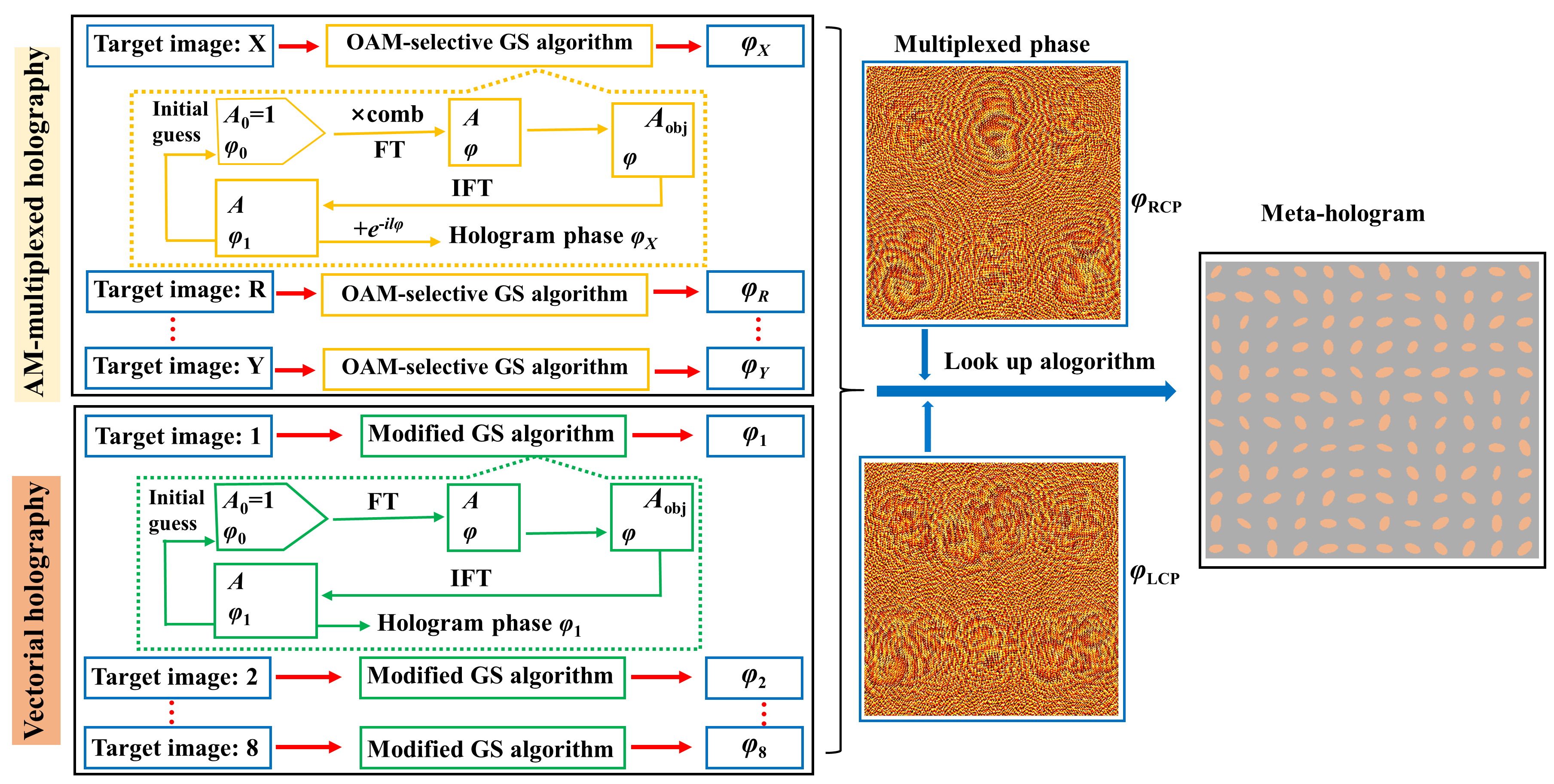


Fig. S6. Phase retrieval process of the AM meta-hologram. FT: Fresnel transformation, IFT: inverse Fresnel transformation.

**6.2 The phase profile of the AM meta-hologram**

For the SOLH, eight capital letters are set as objective images. The corresponding SAM and OAM values of the eight capital letters “X, D, L, E, Y, A, R, F” are |+1, -2>, |+1, -1>, |+1, +1>, |+1, +2>, |-1, -2>, |-1, -1>, |-1, +1> and |-1, +2>, respectively. Hence, the phase profiles of the eight holographic images can be respectively expressed as

$\phi_{X}(|+1,\text{-}2\rangle)=\arg\sum_{j=1}^{j=M} \sum_{k=1}^{k=N} [e^{i(\phi_{Xjk})}|\sigma_{+}\rangle]$ (S14)

$\phi_{D}(|+1,\text{-}1\rangle)=\arg\sum_{j=1}^{j=M} \sum_{k=1}^{k=N} [e^{i(\phi_{Djk})}|\sigma_{+}\rangle]$ (S15)

$\phi_{L}(|+1,+1\rangle)=\arg\sum_{j=1}^{j=M} \sum_{k=1}^{k=N} [e^{i(\phi_{Ljk})}|\sigma_{+}\rangle]$ (S16)

$\phi_{E}(|+1,+2\rangle)=\arg\sum_{j=1}^{j=M} \sum_{k=1}^{k=N} [e^{i(\phi_{Ejk})}|\sigma_{+}\rangle]$ (S17)

$\phi_{Y}(|\text{-}1,\text{-}2\rangle)=\arg\sum_{j=1}^{j=M} \sum_{k=1}^{k=N} [e^{i(\phi_{Yjk})}|\sigma_{-}\rangle]$ (S18)

$\phi_{A}(|\text{-}1,\text{-}1\rangle)=\arg\sum_{j=1}^{j=M} \sum_{k=1}^{k=N} [e^{i(\phi_{Ajk})}|\sigma_{-}\rangle]$ (S19)

$\phi_{R}(|\text{-}1,+1\rangle)=\arg\sum_{j=1}^{j=M} \sum_{k=1}^{k=N} [e^{i(\phi_{Ejk})}|\sigma_{-}\rangle]$ (S20)

$\phi_{F}(|\text{-}1,+2\rangle)=\arg\sum_{j=1}^{j=M} \sum_{k=1}^{k=N} [e^{i(\phi_{Fjk})}|\sigma_{-}\rangle]$ (S21)

where *φ_X_*, *φ_D_*, *φ_L_*, *φ_E_*, *φ_Y_*, *φ_A_*, *φ_R_*, and *φ_F_* are the phase profile of the eight holographic images calculated from the OAM-selective GS algorithm. *M* and *N* are the number of the meta-atoms.

For the SSH, eight objective images of “1, 2, 3, 4, 5, 6, 7, 8” are respectively assigned to the eight polarization channels. The corresponding SoPs are represented as <0, 0|, <0, π|, <0, π/3|, <0, 4π/3|, <0, 2π/3|, <0, 5π/3|, <arcsin(4/5), 0|, and <-arcsin(4/5), π| respectively. These required SoPs at an arbitrary spatial position is the superposition of the two spin eigenstates. Hence, the phase profiles of the eight holographic images can be respectively expressed as

$\phi_{1}=\arg\sum_{j=1}^{j=M} \sum_{k=1}^{k=N} [2e^{i(\phi_{1jk})}|\sigma_{-}\rangle+2e^{i(\phi_{1jk})}|\sigma_{+}\rangle]$ (S22)

$\phi_{2}=\arg\sum_{j=1}^{j=M} \sum_{k=1}^{k=N} [2e^{i(\phi_{2jk}+\pi/2)}|\sigma_{-}\rangle+2e^{i(\phi_{2jk}-\pi/2)}|\sigma_{+}\rangle]$ (S23)

$\phi_{3}=\arg\sum_{j=1}^{j=M} \sum_{k=1}^{k=N} [2e^{i(\phi_{3jk}+\pi/3)}|\sigma_{-}\rangle+2e^{i(\phi_{2jk}-\pi/3)}|\sigma_{+}\rangle]$ (S24)

$\phi_{4}=\arg\sum_{j=1}^{j=M} \sum_{k=1}^{k=N} [2e^{i(\phi_{4jk}+2\pi/3)}|\sigma_{-}\rangle+2e^{i(\phi_{4jk}-2\pi/3)}|\sigma_{+}\rangle]$ (S25)

$\phi_{5}=\arg\sum_{j=1}^{j=M} \sum_{k=1}^{k=N} [2e^{i(\phi_{5jk}+\pi/6)}|\sigma_{-}\rangle+2e^{i(\phi_{5jk}-\pi/6)}|\sigma_{+}\rangle]$ (S26)

$\phi_{6}=\arg\sum_{j=1}^{j=M} \sum_{k=1}^{k=N} [2e^{i(\phi_{6jk}+5\pi/6)}|\sigma_{-}\rangle+2e^{i(\phi_{6jk}-5\pi/6)}|\sigma_{+}\rangle]$ (S27)

$\phi_{7}=\arg\sum_{j=1}^{j=M} \sum_{k=1}^{k=N} [3e^{i(\phi_{7jk})}|\sigma_{-}\rangle+e^{i(\phi_{7jk})}|\sigma_{+}\rangle]$ (S28)

$\phi_{8}=\arg\sum_{j=1}^{j=M} \sum_{k=1}^{k=N} [e^{i(\phi_{8jk}+\pi)}|\sigma_{-}\rangle+3e^{i(\phi_{8jk})}|\sigma_{+}\rangle]$ (S29)

where *φ*_1_, *φ*_2_, *φ*_3_, *φ*_4_, *φ*_5_, *φ*_6_, *φ*_7_, and *φ*_8_ are the phase profile of the eight holographic images calculated from the modified GS algorithm.

Therefore, according to the harmonic strategy, the final phase profiles are endowed to the two spin eigenstates and can be respectively expressed as

$\begin{matrix} \phi_{\text{RCP}}=\arg\sum_{j=1}^{j=M} \sum_{k=1}^{k=N} a\cdot[e^{i(\phi_{Yjk})}+e^{i(\phi_{Ajk})}+e^{i(\phi_{Rjk})}+e^{i(\phi_{Fjk})}+2e^{i(\phi_{1jk})}+2e^{i(\phi_{2jk}+\pi/2)} \\ +2e^{i(\phi_{3jk}+\pi/3)}+2e^{i(\phi_{4jk}+2\pi/3)}+2e^{i(\phi_{5jk}+\pi/6)}+2e^{i(\phi_{6jk}+5\pi/6)}+3e^{i(\phi_{7jk})}+e^{i(\phi_{8jk}+\pi)}] \end{matrix}$ (S30)

$\begin{matrix} \phi_{L\text{CP}}=\arg\sum_{j=1}^{j=M} \sum_{k=1}^{k=N} a\cdot[e^{i(\phi_{Xjk})}+e^{i(\phi_{Djk})}+e^{i(\phi_{Ljk})}+e^{i(\phi_{Ejk})}+2e^{i(\phi_{1jk})}+2e^{i(\phi_{2jk}-\pi/2)} \\ +2e^{i(\phi_{3jk}-\pi/3)}+2e^{i(\phi_{4jk}-2\pi/3)}+2e^{i(\phi_{5jk}-\pi/6)}+2e^{i(\phi_{6jk}-5\pi/6)}+e^{i(\phi_{7jk})}+3e^{i(\phi_{8jk})}] \end{matrix}$ (S31)

where $a=\sqrt{1/n}$represents the normalized electric field intensity (where *n* is the number of the total channels), which is utilized to to achieve equal energy in every channel. Then, using the Eqs. (3-5) in the main text, one can endow these phase profiles to the metasurface by elaborately designing three parameters: *δ_x_*(*x,y*), *δ_y_*(*x,y*) and *θ*(*x,y*), which are finally transformed to the geometric parameters(*L_x_*, *L_y_* and *θ*(*x,y*)) of the meta-atoms.

**7. Numerical and experimental results of the 16 bits AM meta-hologram**

Utilizing the setup shown in Fig. 3c, we characterize the SOL holographic images of the designed meta-hologram. Figure S7 shows the generated incident OAM beams with topological charges of *l* = 0, *l* = ±1, and *l* = ±2, respectively. For the designed AM holography, the size of the incident OAM beams must match the size of the meta-hologram sample. To achieve this goal, the generated OAM beam from the spatial light modulator (SLM) is focused by a lens (see the measurement system in Fig. 3c in the main text). The incident XLP light is treated as a special vortex beam with topological charge *l* = 0. Here, for comparision, the size of the incident XLP light is also modulated by the lens.

Figure S8 shows the numerical and experimental images of intensity distributions generated by the meta-hologram with different incident CP vortex beams.It is observed that the eight objective images can be well reconstructed (with the holographic images appear as Gaussian spots in each pixel) only when a CP vortex beam with specific |σ,*l*> illuminating on the meta-hologram. Otherwise, if the AM of the incident beam is not matching, the resulting holographic images appear as doughnut-shaped intensity distributions in each pixel according to the OAM superposition principle. For optical encryption, such a characteristic can be utilized to protect the required holographic images, with the undesired ones to be the misleading information. For example, if we want to send the message “X” to a specific user. The hacker who want to intercept the message would get a series of misleading information “X, D, L, E” (with the incident OAM does not match). Consequently, eight distinctive SOL holographic images “X, D, L, E, Y, A, R, F” can be well reconstructed through incident CP vortex beams with |+1, -2>, |+1, -1>, |+1, +1>, |+1, +2>, |-1, -2>, |-1, -1>, |-1, +1> and |-1, +2>, respectively.


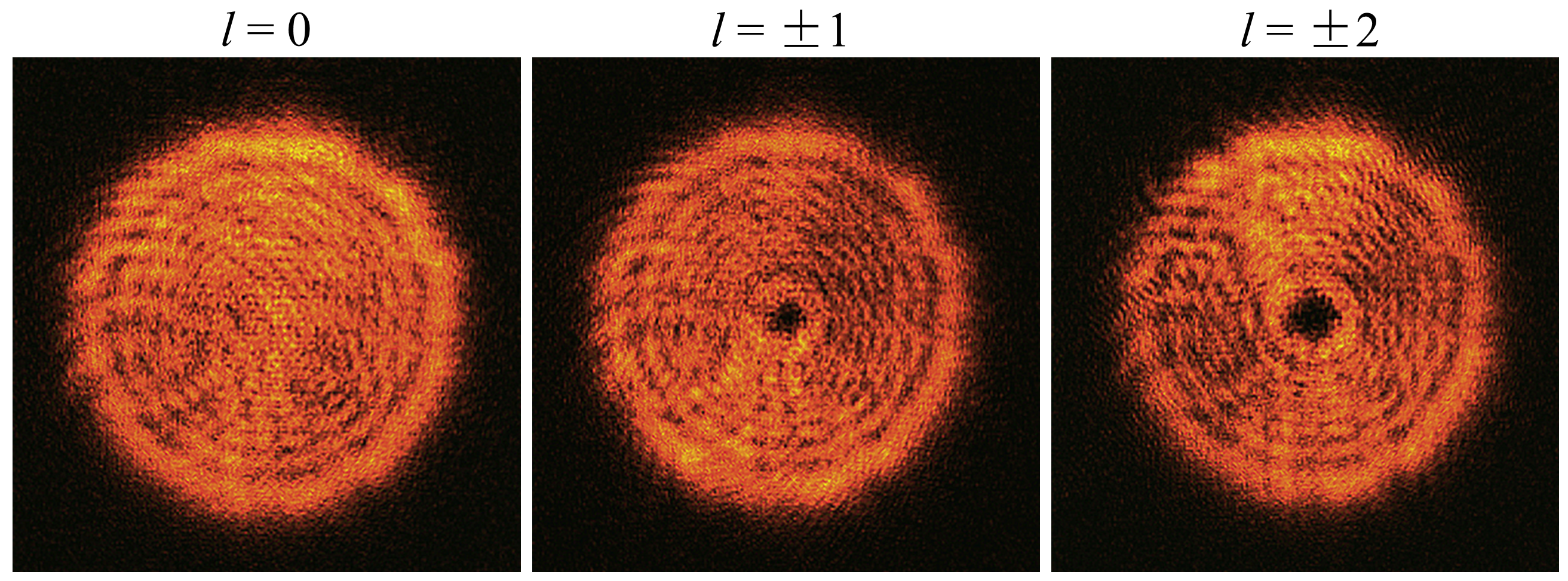


Fig. S7 The generated incident OAM beams with topological charges of *l* = 0, *l* = ±1, and *l* = ±2, respectively. A spatial light modulator (SLM) along with a polarizer and quarter waveplate (QWP) pair are used to generate required OAM beams.


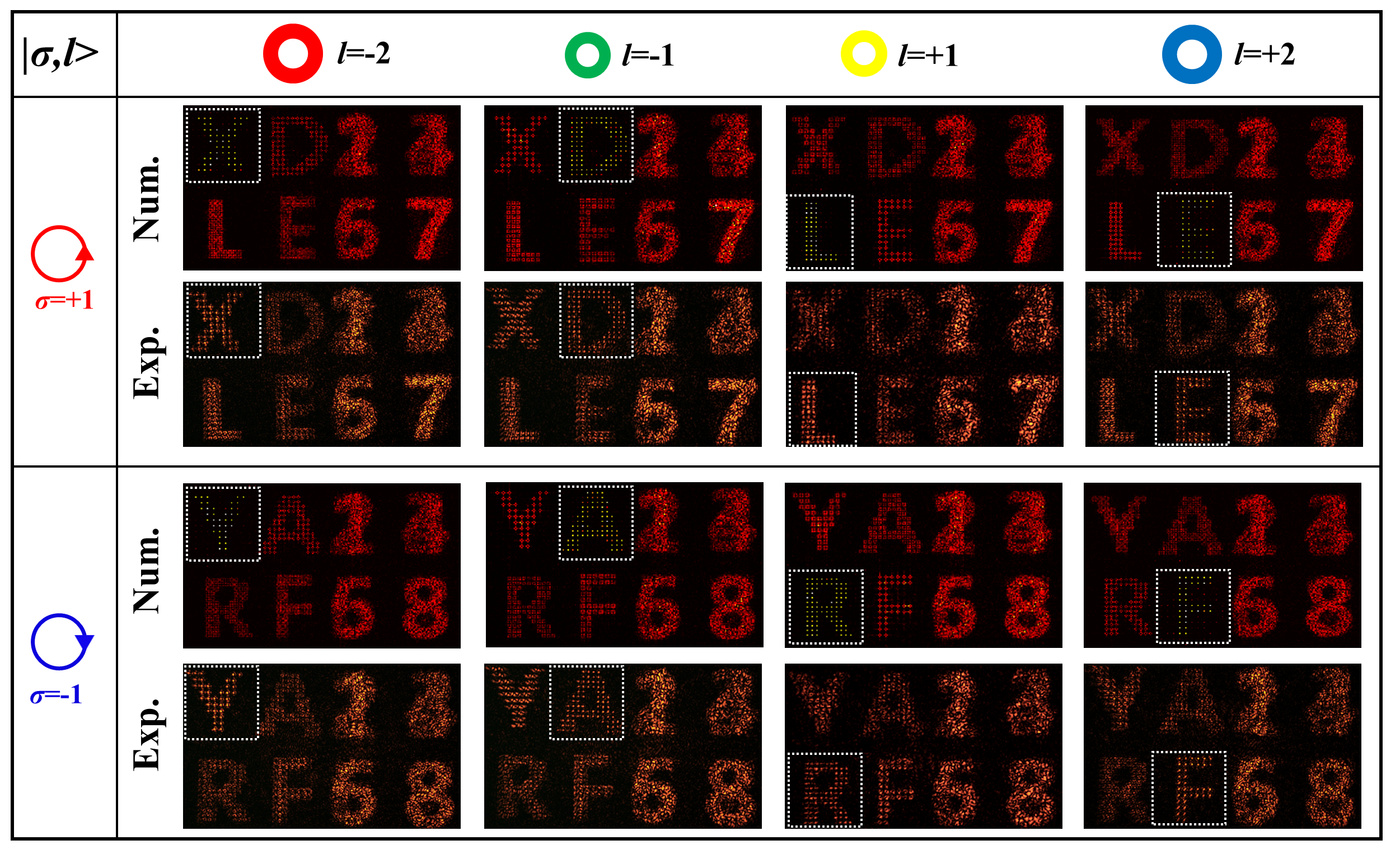


Fig. S8 Numerical and experimental reconstruction of the eight distinctive SOL holographic images through incident CP vortex beams with specific |*σ*, *l*>. The white dashed boxes show the correctly reconstructed SOL holographic images.

Then, we characterize the SS holographic images of the designed meta-hologram by utilizing the same setup shown in Fig. 3c. For generating a XLP Gaussian incident light, the QWP between the SLM and the meta-hologram is removed. In addition, for the extracting of the LP states, the QWP before the CCD is removed. Figure S8 shows the numerical and experimental images of intensity distributions generated by the meta-hologram with a XLP Gaussian incident beam, and simultaneously extracting the specific SoP. Here, two of the eight SS holograhic images (assigned with orthogonal SoP) are elaborately set to superposition with each other, resulting in four superposition images. As shown in Fig. S9, the image with certain encoded SoP can be extracted without any cross-talk, and the other images will coexist with inevitable cross-talk. Such a characteristic can be utilized to protect the required holographic images as well, with the other images to be the misleading information. As a result, eight distinctive SS holographic images “1, 2, 3, 4, 5, 6, 7, and 8” can be well reconstructed by extracting the specific SoP:  <0,0|, <0,π|, <0,π/3|, <0,4π/3|, <0,2π/3|, <0,5π/3|, <arcsin(4/5),0|, and <-arcsin(4/5), π|, respectively. For the designed AM meta-hologram with 16-bit independent channels, the performance of the reconstructed holographic images is not as good as that with only several operating channels. It can be observed that the experimental results show well agreement with the numerical ones. The discrepancy between the two results can be attributed to the fabrication imperfection including etching dose, roughness of surface, and deformation of shapes and the experimental measurement error.


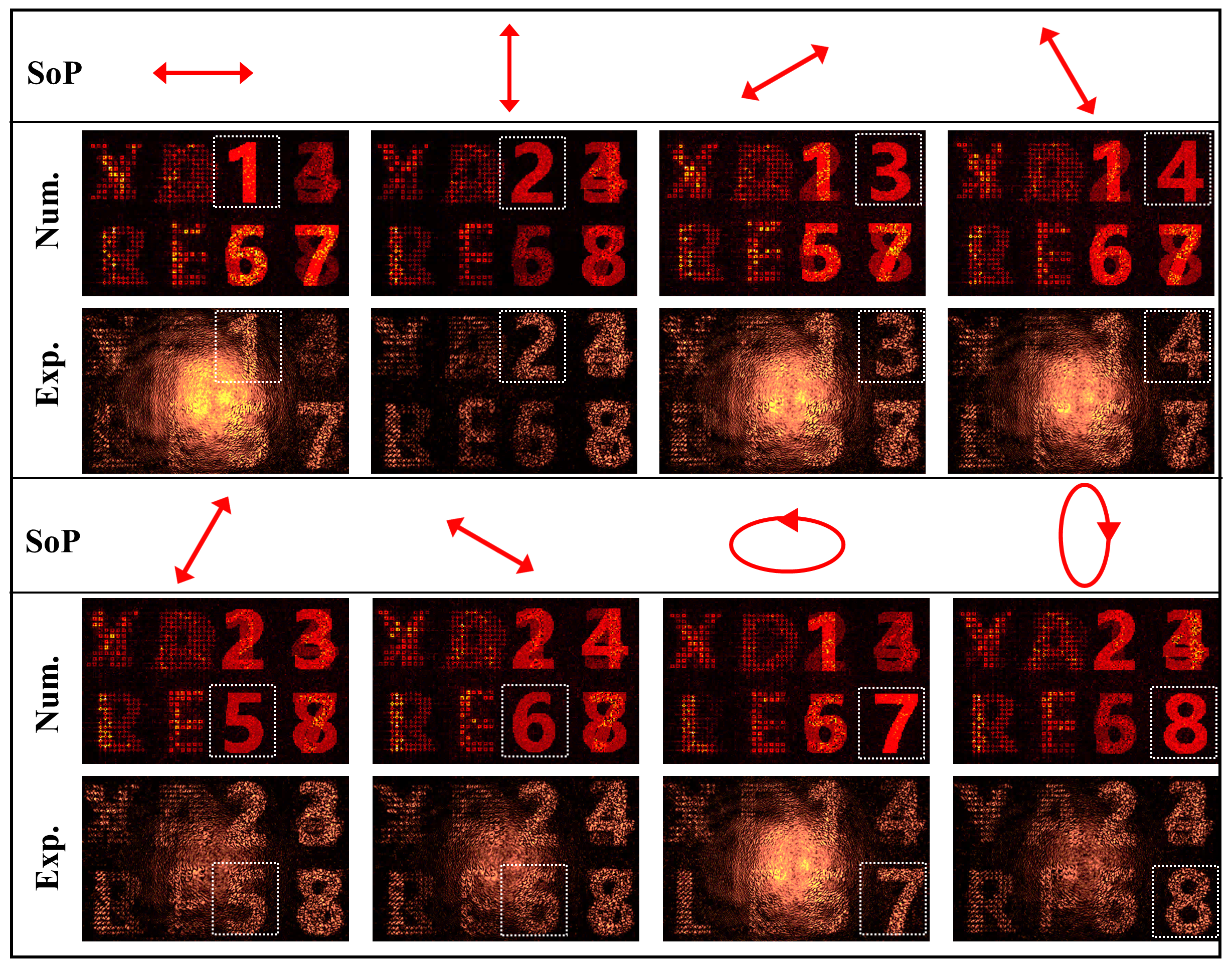


Fig. S9. Numerical and experimental reconstruction of the eight distinctive SS holographic images with LP Gaussian incident beam, and simultaneously extracting the specific SoPs. The white dashed boxes show the correctly extracted SS images.


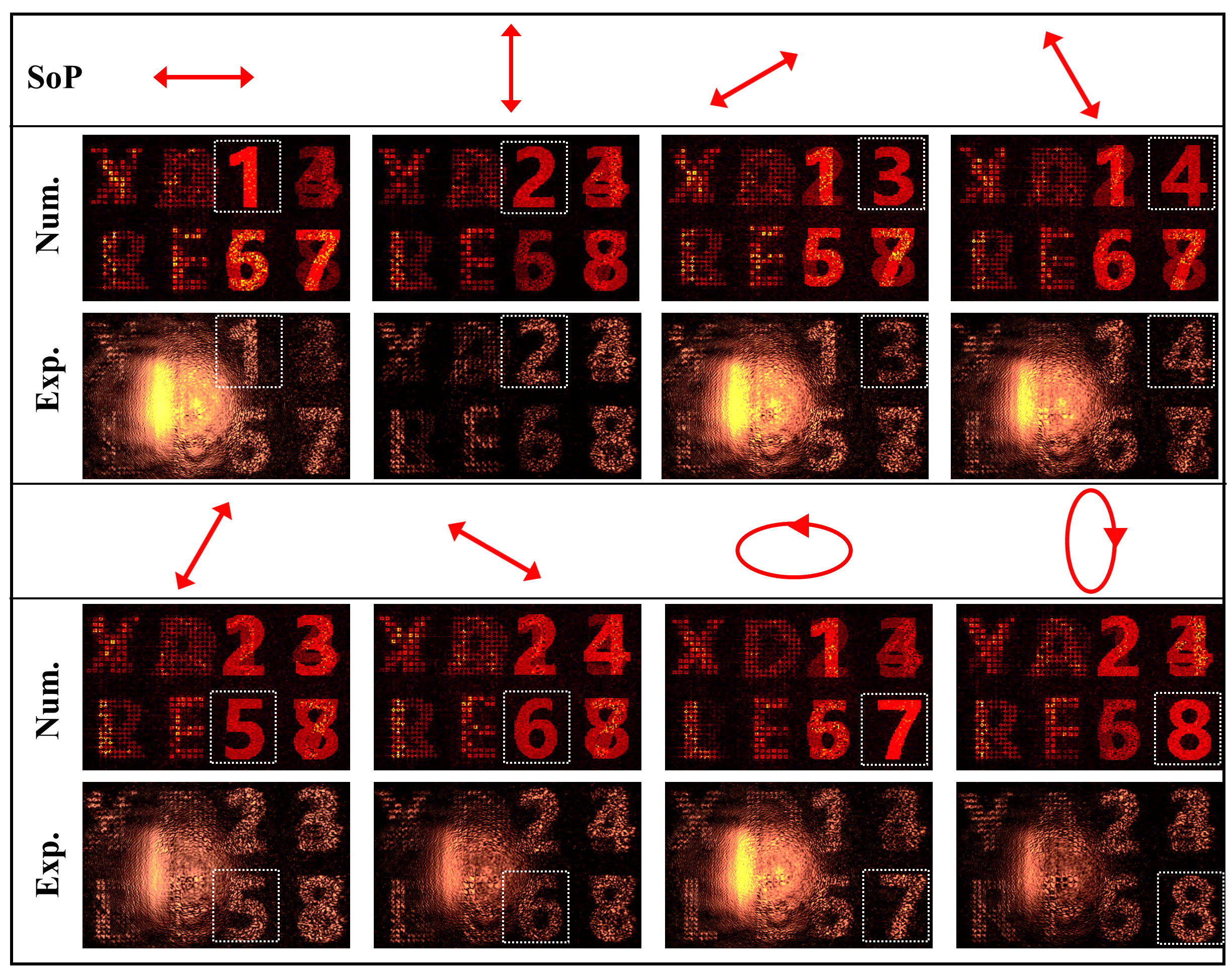


Fig. S10. Numerical and experimental reconstruction of the eight distinctive SS holographic images with LP Gaussian incident beam, and simultaneously extracting the specific SoPs. The white dashed boxes shows the correctly extracted SS images. The incident light deviates to the left from the center of the meta-hologram.

For the SOLH with incident CP vortex beams, we used a polarizer and QWP pair to extract the required CP vortex components (with the undesired components are totally blocked). As a result, the unmodulated zero-level diffracted light completely disappears in both simulated and experimental results, as shown in Fig. S8. For the SSH, we use a plane wave in the simulation and hence the unmodulated zero-level diffracted light is not exhibited in the simulated results. In the experimental measurement, the incident XLP light is focused by a lens. As shown in Fig. S9, only when we extract the *y*-linearly polarized component, the unmodulated zero-level diffracted light can be totally blocked. Otherwise, the unmodulated zero-level diffracted light cannot be totally blocked and is coexist with the holographic images. To reduce the influence of unmodulated zero-level diffracted light, we set the incident light deviates from the center of the meta-hologram. The corresponding numerical and experimental results are shown in Fig. S10, from which we can extract the needed images with little influence of unmodulated zero-level diffracted light. The unmodulated zero-level diffracted light would severely affect the performance of holographic images for displaying-related applications. Here, for the application of optical encryption in our manuscript, these results is acceptable since we can get the required patterns of the objective numbers.

**8. Numerical results of a 32 bits AM meta-hologram**

In the main text, we numerically and experimentally demonstrate the using of OAM states ranging from -2 to +2 (with the total number of OAM states is 4) for holographic multiplexing. For massive information transmission in practical applications, the higher order OAM states must be taken into consideration. To further verify the design principle, we numerically design an AM meta-hologram with 32 multiplexing channels. Here, OAM states with topological charges ranging from 1 to 12 are taken into consideration (with the total number of OAM states is larger than 10). Twenty-four capital letters (ranging from ‘A’ to ‘X’) are set as target images of the SOLH. The corresponding SAM and OAM values of the 24 capital letters are represented as |+1, 1>, |+1, 2>, |+1, 3>, |+1, 4>, |+1, 5>, |+1, 6>, |+1, 7>, |+1, 8>, |+1, 9>, |+1, 10>, |+1, 11>, |+1, 12>, |-1, 1>, |-1, 2>, |-1, 3>, |-1, 4>, |-1, 5>, |-1, 6>, |-1, 7>, |-1, 8>, |-1, 9>, |-1, 10>, |-1, 11>, and |-1, 12>, respectively. The objective images of the SSH are chose as eight Arabic numbers ranging from ‘1’ to ‘8’, which are assigned with eight distinct SoPs represented as <2*χ*, 2*ψ*|. The corresponding SoPs of the eight images are <0, 0|, <0, π|, <0, π/3|, <0, 4π/3|, <0, 2π/3|, <0, 5π/3|, <arcsin(4/5), 0|, and <-arcsin(4/5), π|, respectively.

Figure. S11 shows the numerical reconstructed 12 distinctive SOL holographic images under incident LCP vortex beams with specific |*σ*, *l*>. In the design process, the sampling constants for different OAM are set as an identical value, which corresponds to the spatial frequency distribution in the image plane of OAM beam with *l* = 12. This is reasonable for the fact that if the spatial frequency distribution of larger OAM mode is not overlayed, and the spatial frequency distributions of the samller ones will definitely not overlayed. The same design strategy has been utilized for demonstrting multiple channel OAM-multiplexed holography [4]. The white dashed boxes show the correctly reconstructed SOL holographic images. It can be observed that twelve distinctive SOL holographic images “A, B, C, D, E, F, G, H, I, J, K, L” can be well reconstructed (with each pixel appear as Gaussian spots) through incident CP vortex beams with |+1, 1>, |+1, 2>, |+1, 3>, |+1, 4>, |+1, 5>, |+1, 6>, |+1, 7>, |+1, 8>, |+1, 9>, |+1, 10>, |+1, 11>, and |+1, 12>, respectively. Similarly, Fig. S12 shows the numerical reconstructed 12 distinctive SOL holographic images under incident RCP vortex beams with specific |*σ*, *l*>. Consequently, twelve distinctive SOL holographic images “M, N, O, P, Q, R, S, T, U, V, W, X” can be well reconstructed through incident CP vortex beams with |-1, 1>, |-1, 2>, |-1, 3>, |-1, 4>, |-1, 5>, |-1, 6>, |-1, 7>, |-1, 8>, |-1, 9>, |-1, 10>, |-1, 11>, and |-1, 12>, respectively.

Figure S13 shows the numerical results of intensity distributions generated by the meta-hologram with a XLP incident beam, and simultaneously extracting the specific SoP. Here, two of the eight SS holograhic images (assigned with orthogonal SoP) are elaborately set to superposition with each other, resulting in four superposition images. As shown in Fig. S13, the image with certain encoded SoP can be extracted without any cross-talk, and the other images will coexist with inevitable cross-talk. As a result, eight distinctive SS holographic images “1, 2, 3, 4, 5, 6, 7, and 8” can be well reconstructed by extracting the specific SoP: <0, 0|, <0, π|, <0, π/3|, <0, 4π/3|, <0, 2π/3|, <0, 5π/3|, <arcsin(4/5), 0|, and <-arcsin(4/5), π|, respectively.

To clearly demonstrate the results of the designed AM meta-hologram with 32 independent channels, the desired holographic images are shown in Fig. S14. The results are comparable to that of the AM meta-hologram with 16 multiplexing channels. Although with reduced signal to noise ratio, the desired holographic images are well reconstructed. For the SOLH, the capital letters are well reconstructed(with each pixel appear as Gaussian spots) under incident beam with correct SAM and OAM values. Moreover, eight SS holographic images of Arabic numbers from 1 to 8 can also be well reconstructed by extracting the specific SoPs. Therefore, we can conclude that the design concept can still work well for the AM meta-hologram with up to 32 multiplexing channels.

**
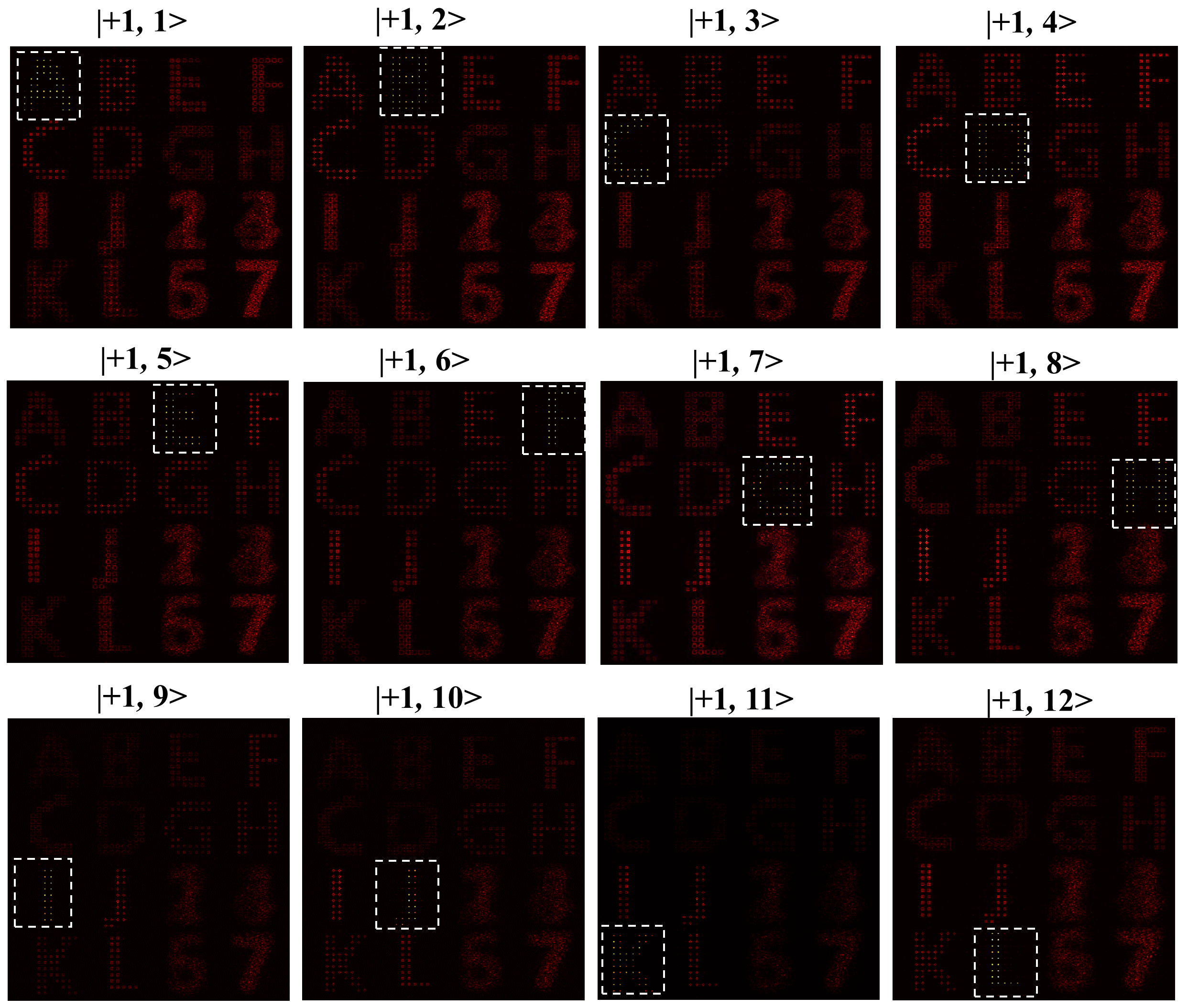
**

Fig. S11 Numerical reconstruction of the 12 distinctive SOL holographic images under incident LCP vortex beams with specific |*σ*, *l*>. The white dashed boxes show the correctly reconstructed SOL holographic images.

**
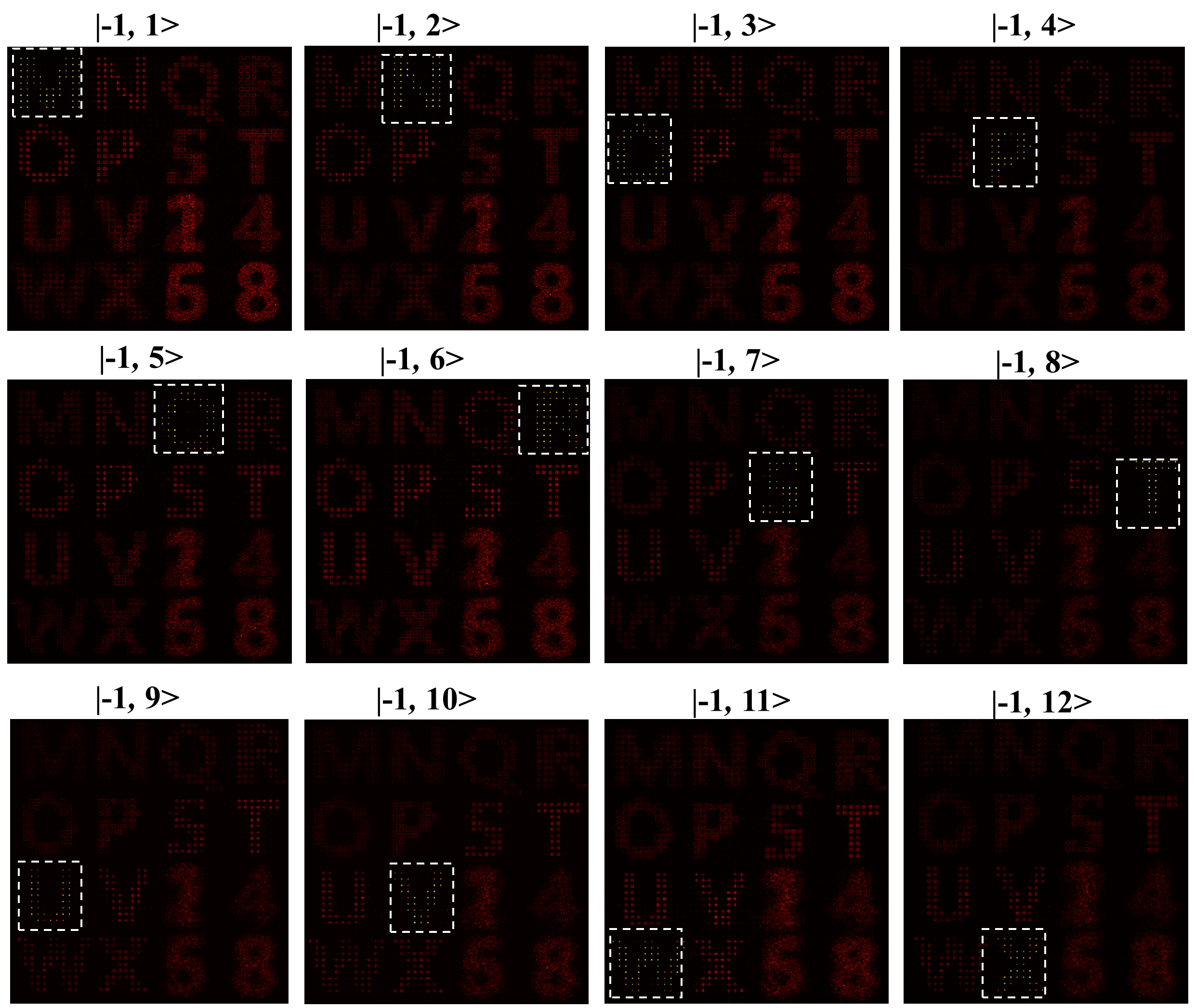
**

Fig. S12 Numerical reconstruction of the 12 distinctive SOL holographic images through incident RCP vortex beams with specific |*σ*, *l*>. The white dashed boxes show the correctly reconstructed SOL holographic images.

**
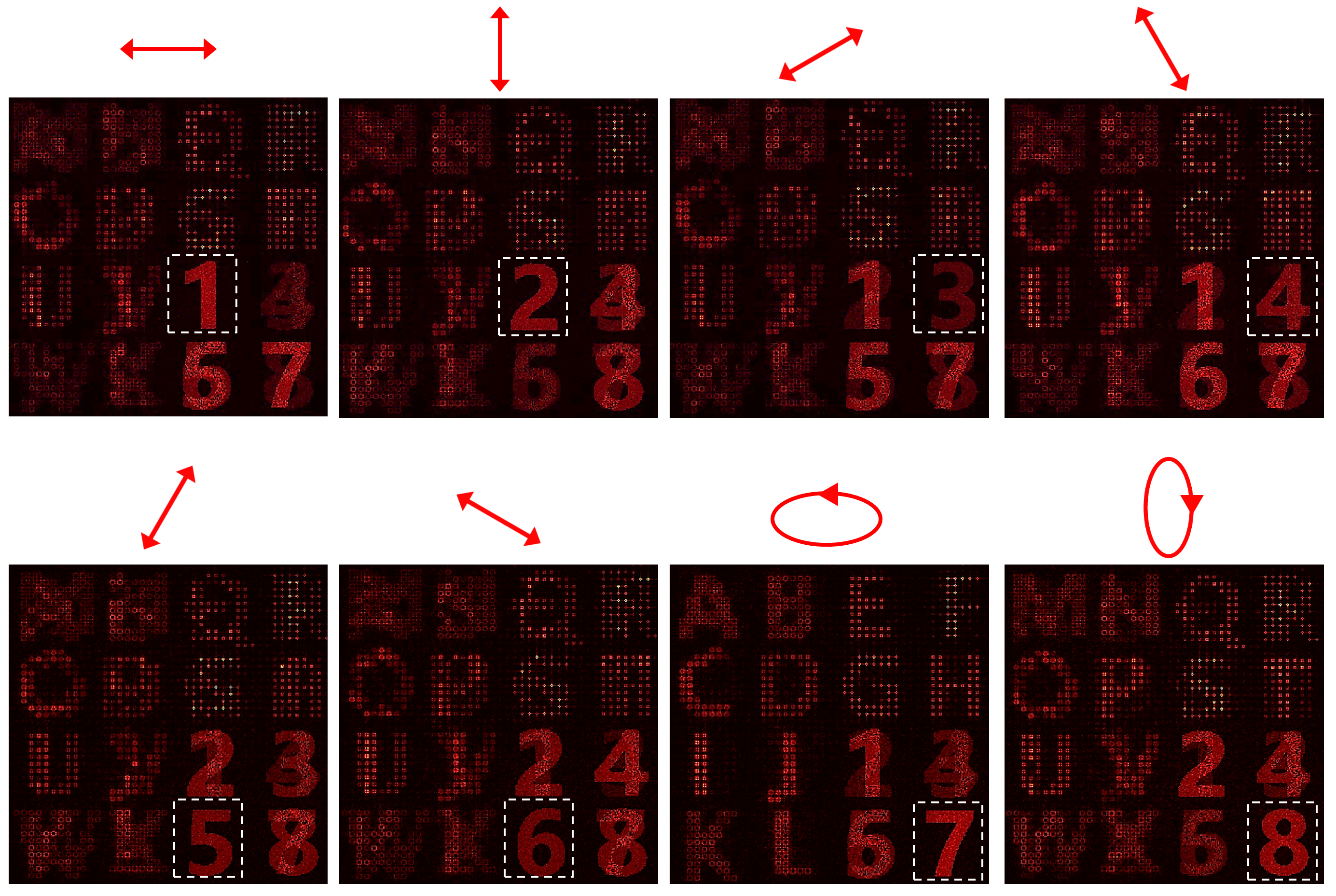
**

Fig. S13. Numerical reconstruction of the 8 distinctive SS holographic images with LP Gaussian incident beam, and simultaneously extracting the specific SoPs. The white dashed boxes shows the correctly extracted SS images.

**
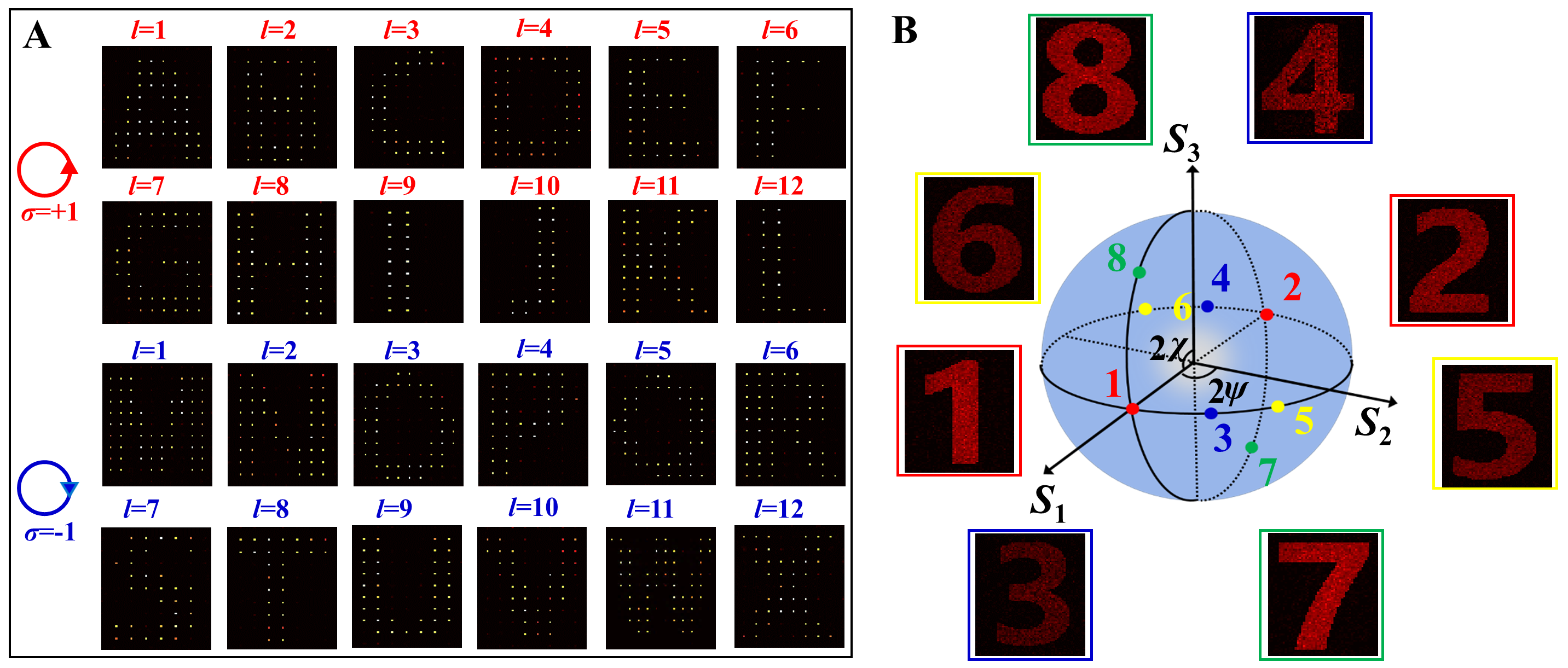
**

Fig. S14. (A) Numerical reconstruction of the twenty-four distinctive SOL holographic images through incident CP vortex beams with specific |*σ*, *l*>. (B) Numerical reconstruction of the eight distinctive SS holographic images through a XLP incident light and extracting certain SoPs via utilizing specific analyzers. The corresponding SoPs of the 8 holographic images indicate by eight points on the PS.

**References**

[1] J. P. B. Mueller, N. A. Rubin, R. C. Devlin, B. Groever, and F. Capasso, “Metasurface Polarization Optics: Independent Phase Control of Arbitrary Orthogonal States of Polarization,” Phys. Rev. Lett. 118(11), 113901 (2017).

[2] G. Ding, K. Chen, X. Luo, J. Zhao, T. Jiang, and Y. Feng, “Dual-helicity decoupled coding metasurface for independent spin-to-orbital angular momentum conversion,” Phys. Rev. A 11(4): 044043 (2019).

[3] V. V. Kotlyar, S. N. Khonina, A. A. Kovalev, and V. A. Soifer, “Diffraction of a plane, finite-radius wave by a spiral phase plate,” Opt. Lett. 31, 1597-1599 (2006).

[4] X. Fang, H. Ren, and M. Gu, “Orbital angular momentum holography for high-security encryption,” Nature Photonics 14, 102-108 (2020).
